# Biologics for atopic diseases: Indication, side effect management, and new developments 

**DOI:** 10.5414/ALX02197E

**Published:** 2021-01-05

**Authors:** Uta Jappe, Hendrik Beckert, Karl-Christian Bergmann, Askin Gülsen, Ludger Klimek, Sandra Philipp, Julia Pickert, Michèle M. Rauber-Ellinghaus, Harald Renz, Christian Taube, Regina Treudler, Martin Wagenmann, Thomas Werfel, Margita Worm, Torsten Zuberbier

**Affiliations:** 1 Research Group Clinical and Molecular Allergology of the Research Center Borstel, Airway Research Center North (ARCN), Member of the German Center for Lung Research (DZL),; 2 Interdisciplinary Allergy Outpatient Clinic, Medical Clinic III, University of Lübeck,; 3 Department of Pulmonary Medicine, University Hospital Essen – Ruhrlandklinik, Essen,; 4 Charité – Universitätsmedizin Berlin, corporate member of Freie Universität Berlin, Humboldt-Universität zu Berlin, and Berlin Institute of Health,; 5 Center for Rhinology and Allergology, Wiesbaden,; 6 Dermatology practice Dr. Markus Friedrich/Dr. Sandra Philipp, Oranienburg,; 7 Department of Dermatology and Allergology, University Hospital Gießen and Marburg, Marburg site,; 8 Experimental Dermatology and Allergology, Justus Liebig University Giessen,; 9 Department of Medicine, Institute of Laboratory Medicine and Pathobiochemistry – Molecular Diagnostics, Member of the German Centre for Lung Research (DZL), Philipps-University, Marburg,; 10 Leipzig Comprehensive Allergy Center LICA-CAC, Department of Dermatology, Venereology and Allergology, University of Leipzig,; 11 Department of Otorhinolaryngology, HNO-Klinik, Universitätsklinikum Düsseldorf, Düsseldorf,; 12 Division of Immunodermatology and Allergy Research, Department of Dermatology and Allergy, Hannover Medical School, Hannover,; 13 Dermatology, Venerology and Allergology, Charité – Universitätsmedizin Berlin, corporate member of Freie Universität Berlin, Humboldt-Universität zu Berlin, and Berlin Institute of Health, Berlin,; 14 Department of Dermatology and Allergy, Charité Universitätsmedizin, Berlin, Humboldt-Universität zu Berlin, and Berlin Institute of Health, Comprehensive Allergy Center, Berlin, Germany

**Keywords:** allergy diagnostics, anti drug antibodies, COVID 19, hypersensitivity reactions, vaccination, food allergy, off-label-use, pregnancy

## Abstract

With the advent of biologicals, more and more therapeutics are available that specifically address specific switch points in the pathomechanism of immunologically dominated diseases. Thus, the focus of diagnostics and therapy (precision medicine) is more on the individual disease characteristics of the individual patient. Regarding the different phenotypes of atopic diseases, severe asthma was the first entity for which biologicals were approved, followed by urticaria, and finally atopic dermatitis and chronic rhinosinusitis with nasal polyps. Experience in the treatment of severe bronchial asthma has shown that the intensity of the response to biological therapy depends on the quality of clinical and immunological phenotyping of the patients. This also applies to different diseases of the atopic form, as patients can suffer from several atopic diseases at the same time, each with different characteristics. Biologics are already emerging that may represent a suitable therapy for allergic bronchial asthma, which often occurs together with severe neurodermatitis, and chronic rhinosinusitis with nasal polyps. In practice, however, the question of possible combinations of biologicals for the therapy of complex clinical pictures of individual patients is increasingly arising. In doing so, the side effect profile must be taken into account, including hypersensitivity reactions, whose diagnostic and logistical management must aim at a safe and efficient therapy of the underlying disease. Increased attention must also be paid to biological therapy in pregnancy and planned (predictable) vaccinations as well as existing infections, such as SARS-CoV-2 infection. Before starting a biological therapy, the immune status should be checked with regard to chronic viral and bacterial infections and, if necessary, the vaccination status should be refreshed or missing vaccinations should be made up for before starting therapy. Currently, reliable data on the effect of biologicals on the immunological situation of SARS-CoV-2 infection and COVID-19 are not available. Therefore, research and development of suitable diagnostic methods for detection of immunologically caused side effects as well as detection of potential therapy responders and non-responders is of great importance.

## Introduction 

The increasing elucidation of pathomechanisms of oncological and inflammatory diseases at the cellular and molecular level and the realization that the focus of diagnostics and therapy must no longer be on the disease itself but on the individual patient (precision medicine) has led to the development of targeted therapeutics in recent years (target treatments). The so-called biologicals are substances that imitate actors of the human organism/immune system and can modulate the immune system in different ways. 

The biologics that are the subject of this review are mainly composed of active ingredients of the following substance groups: monoclonal antibodies (mAB), cytokines, and fusion proteins. They act specifically via binding to receptors (activation or inhibition) or via the complexation of active structures with the aim of cancelling their effect (cytokine and antibody inhibitors). 

The mAB can be chimera, i.e., they consist of human and murine parts. However, due to their relatively high immunogenicity (< 50 – 75% human) and to increase efficiency, more and more humanized or human mAB have been produced and approved. 

Fusion proteins are essentially constructs consisting of a soluble protein and an IgG1 antibody fragment (Fc-part) and can thus represent a ligand or a receptor, depending on the construction design, which has a high affinity to the corresponding target. 

The fact that biologicals are constructed according to their target structures should not hide the fact that the respective mechanisms of action have not yet been elucidated and understood in detail. The immune-modulating properties are partly responsible for undesired immunological reactions like hypersensitivity reactions, induction of autoimmune diseases, and immunodeficiency, and for some biologicals also non-immunological side effects have become known, e.g., the acneiform exanthema under cetuximab. Among the immunological side effects, the cytokine release syndrome (“cytokine storm”) and anaphylaxis are among the most feared. 

Inflammatory diseases already successfully treated with biologicals include psoriasis (and psoriatic arthritis), rheumatoid arthritis, multiple sclerosis, inflammatory bowel disease, chronic urticaria, asthma, chronic rhinosinusitis with nasal polyps, and atopic dermatitis. 

In the following, the biological therapy of atopic diseases is described, and new approvals or expected approvals are briefly described. 

## Biologics for the treatment of bronchial asthma 

Biologics are used to treat patients with certain phenotypes of severe allergic asthma. Omalizumab with this indication was approved in 2005. Other biologics are now available for the treatment of patients with certain forms of asthma. These include antibodies that block IL-5 (mepolizumab, reslizumab), the IL-5 receptor (benralizumab), or the IL-4 receptor alpha chain (dupilumab) [1]. 

The applications of these biologics are currently reserved for patients with severe asthma. However, there is no single definition of severe asthma. Several different approaches have been published to define the patient group “severe asthma”. The main principle of the definitions is the presence of uncontrolled asthma despite high-dose inhaled anti-inflammatory therapy (inhaled corticosteroids) in combination with another controller (e.g., long-acting beta-2 sympathomimetics). Evaluations of insurance and health insurance data suggest that this definition affects ~ 3 – 4% of patients with asthma [2, 3]. Uncontrolled asthma can be objectified by questionnaires (Asthma Control Test or Asthma Control Questionnaire), the presence of acute worsening (exacerbations), inpatient treatment due to exacerbation, and impaired lung function. It is important to distinguish between patients with “difficult-to-treat” asthma and patients with “severe” asthma [4]. In the majority of patients whose asthma is not controlled despite high-dose ICS therapy, factors can be identified that are the cause of poor symptom control. These factors include inadequate drug intake (e.g., inadequate inhalation technique, lack of adherence), unidentified or untreated comorbidities (e.g., sleep apnea, obesity, reflux, chronic rhinosinusitis), or persistent trigger factors (allergen sources in the environment). In these patients, the management and correction of these factors is of primary importance, and in a large proportion of patients, control of the disease can be achieved without the use of biologicals. If patients remain symptomatic despite evaluation and treatment of the above-mentioned factors, severe asthma is present. These patients should then be evaluated for the possible use of a monoclonal antibody. 

National and international guidelines clearly recommend that any antibody therapy [5, 6] should be preferred to treatment with systemic corticosteroids. Prolonged and repeated treatment with systemic corticosteroids also leads to side effects in patients with asthma [3, 7]. 

The diagnosis asthma includes patients with different clinical manifestations and different immunological alterations. Therefore, good clinical and immunological phenotyping is necessary to identify patients with a high probability of a response to biological treatment. For the phenotypes severe allergic asthma, asthma with eosinophilic inflammatory response and asthma with type 2 inflammation, antibodies are available. Please note that these phenotypes cannot always be clearly separated from each other, but partly overlap considerably. 

Omalizumab has been approved for patients with severe allergic asthma since 2005. In these patients, treatment with omalizumab can contribute to a reduction in the rate of exacerbation, an improvement in symptoms and quality of life and an improvement in lung function. Omalizumab can also reduce the need for systemic steroid therapy [8]. Recent data also show that omalizumab is effective regardless of the type of inflammation detected. A reduction of acute exacerbations has been shown in patients with and without eosinophilic inflammation [9]. 

In patients with severe asthma and an inflammatory response with eosinophilic granulocytes, three antibodies against the cytokine itself (IL-5) or against the α-chain of the human IL-5 receptor (IL-5Rα) have now been developed and approved for treatment. Mepolizumab and reslizumab are approved as anti-IL-5 antibodies. Clinical studies on these preparations have shown that patients with the detection of an increased number of eosinophilic granulocytes in peripheral blood under mepolizumab experience a significant reduction in exacerbations, an improvement in asthma control, and also an improvement in FEV1 [10]. Similar results have been reported for benralizumab [11], which binds to IL-5Rα as an antibody, and patients with severe asthma and eosinophilia experience a reduction in exacerbations, improvement in symptoms and quality of life, and a slight improvement in lung function [12]. 

Particularly important are the effects of mepolizumab and benralizumab in patients who require treatment with a systemic steroid due to their asthma. In controlled studies, it was shown that after administration of anti-IL-5 or anti-IL-5Rα, a reduction of systemic steroids or complete discontinuation was possible in patients with steroid-dependent asthma [10, 13]. Despite the reduced steroid dose, there were fewer exacerbations in the treated groups. Since treatment with systemic steroids can have considerable side effects, these results are of considerable relevance. Dupilumab is also approved as another antibody for patients with severe asthma. Dupilumab binds to the alpha chain of the interleukin-4 receptor (IL-4Rα) and thereby inhibits the binding of IL-4 and IL-13 to the respective receptor. Dupilumab has also been shown to reduce exacerbations, improve quality of life and lung function in patients with uncontrolled asthma in whom eosinophilic inflammation or elevated levels of nitric oxide (FeNO) in exhaled air have been detected [14]. Dupilumab has also been shown to significantly reduce the dose of systemic steroids in patients treated with systemic steroids on a long-term basis; in some cases, it was even possible to discontinue them completely [14]. 

Omalizumab, mepolizumab, benralizumab, and dupilumab have now been approved for self-administration. Since anaphylactic reactions to biologicals can occur even after months of successful application [15], self-injection at home is a risk that should not be underestimated. 

Treatment should be started by physicians experienced with severe asthma. The effectiveness of the treatment with biologicals should be evaluated after 4 months. If the response is not clearly detectable, the evaluation phase can be extended to 12 months. After the start of treatment with biologicals, the previous inhaled and oral asthma therapy should be maintained for at least 4 weeks and only after this time should it be reduced if necessary under close assessment of asthma control. 

It should be noted that all biologicals are an add-on therapy and are not approved for monotherapy. In a number of patients, however, the use of biologicals leads to such an improvement in lung function, asthma control test, and symptoms that patients can – and do – completely avoid the further use of inhaled steroids and long-acting beta-mimetics. Without there being any national or international recommendation for these situations, in these cases, an extension of the injection intervals should be considered. For omalizumab, it has already been described that after reaching a controlled stage, it is possible to significantly extend the injection intervals [16], while a reduction or discontinuation of the biological agent usually led to renewed deterioration. For the other biologics, this procedure also appears possible in individual cases, although not corresponding to the approved description of indications. 

## Biologics for the treatment of urticaria 

In urticaria, one biological agent, omalizumab, is currently approved for therapy, and a number of others are currently undergoing clinical trials. 

Urticaria is defined as a disease with the sudden appearance of wheals, angioedema, or both. Chronic urticaria is defined as a disease with a course of more than 6 weeks. It is divided into chronic spontaneous urticaria and chronic inducible urticaria. The latter in turn has various subforms, partly triggered by physical stimuli, e.g., cold urticaria, partly by other exogenous factors, e.g., cholinergic urticaria. In accordance with current international guidelines, all chronic forms of urticaria are treated equally according to one algorithm [17]. In the first stage, this algorithm recommends treatment with a non-sedating antihistamine in the single dose, and in case of non-response, a dose increase up to 4 times the single dose is applied in the second stage. In case of further non-response, the additional administration of omalizumab is recommended in the 3^rd^ stage, and in the 4^th^ stage the administration of cyclosporine A is recommended in case of further non-response. The Urticaria Activity Score (UAS), which has been validated for chronic spontaneous urticaria, has been developed to assess the clinical response of urticaria therapy. Itching is measured on a scale of 0 – 3 and the number of wheals on a scale of 0 – 3. This means that the maximum daily value is 6. Since urticaria fluctuates, for response UAS 7 is calculated, i.e., the sum of the daily values over 1 week. The maximum response therefore is 42. 1 week’s UAS 7 of 6 or less is currently considered sufficient, although the actual treatment goal is being symptom-free according to the guideline. 

The 3^rd^ stage of the algorithm is the administration of omalizumab as an additional therapy to high-dose antihistamine administration. Omalizumab is a humanized monoclonal antibody against IgE. Its efficacy in chronic spontaneous urticaria has been demonstrated in numerous large studies and is 52 – 90% in antihistamine-refractory patients [18, 19, 20, 21]. 

It’s safety profile is also very good. In the clinical trials, the rate of side effects was comparable to placebo. The most commonly reported adverse events included nasopharyngitis, sinusitis, and colds without likely relation to the drug [20, 21, 22, 23]. Anaphylactic reactions have been reported in asthma patients, but these were not observed in the treatment of urticaria, and the drug is now approved as a ready-to-use subcutaneous syringe for self-application. A major advantage of the safety of omalizumab is that no preliminary studies are required, such as the exclusion of tuberculosis in TNF-alpha antagonists and the fact that no antibodies blocking the action of omalizumab have been described. This allows a flexible handling of the drug. The approval documents a fixed dose of 300 mg s.c., which corresponds to two 150-mg syringes, to be administered every 4 weeks. Recent real-life results show, however, that under certain circumstances, if there is no treatment response, it may be appropriate to either shorten the interval or increase the dose [24, 25]. In particular, overweight patients may benefit from an upward dose adjustment. On the other hand, the absence of blocking antibodies allows patients who respond fully to treatment to stop taking the medication after a period of 3 – 6 months without any risk of reducing the effectiveness of the medication when it is reapplied. Although not yet noted in the algorithm in the current guidelines, there is now well-established scientific evidence that in those patients who do not respond to omalizumab 300 mg at 4-weekly intervals, a dose increase to initially 450 mg and possibly also to 600 mg will increase the response rate. A general distinction is made between fast and slow response in different patients. In some patients, the response is almost complete 24 hours after the first dose. Other patients show only a slow improvement of UAS7 over the first 3 months of omalizumab therapy. Although it is not possible to predict with certainty whether a fast or slow response will be observed in individual patients, it is generally true that patients with very low total IgE respond less well or not at all. For those patients who do not respond to omalizumab, the algorithm of the international guideline recommends the administration of cyclosporine A [17]. In practice, however, cyclosporine A can also be combined with omalizumab. 

Omalizumab has revolutionized the treatment of chronic spontaneous urticaria but is also effective in the treatment of chronic inducible urticaria [22, 26]. Study results – or at least case series – are now available for most forms of inducible urticaria. 

Due to the efficacy of omalizumab, the first commercially available anti-IgE antibody, the significance of IgE-antibodies directed against endogenous structures has become more evident. Not only is total IgE elevated on average in patients with urticaria, but anti-dsDNA, anti-thyroid globulin, and anti-thyroid peroxidase IgE are also found in a number of patients [27, 28]. Against this background, further biologics have been developed and are currently in various stages of clinical testing. The most advanced are the phase 3 studies on ligelizumab, a humanized IgG1 antibody directed against the Ce3 domain of IgE. Compared to omalizumab, it shows significantly higher inhibition of IgE binding to the high-affinity IgE receptor but lower inhibition of IgE binding to the low-affinity receptor CD23 [29]. 

## Biologics for the treatment of atopic dermatitis 

The first biological agent approved for the treatment of atopic dermatitis is dupilumab, a recombinantly produced human IgG4 monoclonal antibody. The antibody specifically targets the common IL-4Rα subunit of type 1 and type 2 IL-4 receptors and thus blocks not only interleukin 4 but also interleukin 13 and thus two key cytokines of atopic inflammation. Dupilumab was approved by the European Medicines Agency (EMA) at the end of 2017 for adults with moderate to severe atopic dermatitis after an extensive study program with two successful placebo-controlled phase 3 studies [30], a long-term study over 1 year in which topical corticosteroids were allowed to be used in the comparative arm [31]. In autumn 2019, approval was granted for children from 12 years of age and adolescents, after a placebo-controlled study was successfully completed in this age group as well [32]. A phase 3 study in the age group of 6- to 11-year-old children has been completed, but has not yet led to an extension of the approval [33]. 

The approval of dupilumab for the indication of atopic dermatitis represents a milestone in the treatment of moderate to severe forms of this disease, since apart from corticosteroids, which, according to the guidelines, should only be used as an interventional therapy for a maximum of 3 weeks in adults, until then only cyclosporine for the treatment of atopic dermatitis from the age of 16 had been approved. In the updated AWMF guideline for the systemic treatment of atopic dermatitis, dupilumab was included in the 2020 recommendations [34]. 

The neurodermatitis registry TREATgermany recorded a correspondingly large number of patients with moderate to severe atopic dermatitis who have been treated with the antibody since then, while according to registry data cyclosporine and other “off-label” immunosuppressants have been used significantly less frequently for the indication atopic dermatitis since then [35]. Under “real-life conditions” of the German neurodermatitis registry TREATgermany, the efficacy under treatment with dupilumab in terms of improvement of severity and subjective symptoms was in a similar spectrum as in the previously published phase 3 studies [36]. 

The main side effects of dupilumab occur in the eye, with non-allergic conjunctivitis and other changes in the eye occurring exclusively as a side effect in patients with atopic dermatitis (and not in patients with allergic bronchial asthma or chronic rhinosinusitis with nasal polyps). There are a number of speculations on the pathomechanism, each of which sounds plausible, but which have not been verified to date [37]. Fortunately, most patients who develop (peri-)orbicular changes (~ 10 – 15% of all patients on dupilumab therapy) are able to continue therapy with symptomatic treatment [38]. 

In view of the ongoing SARS-CoV-2 pandemic, two meta-analyses on the frequency of infections under dupilumab therapy are important to show that there has been no increase in systemic infections under therapy with the antibody in controlled studies. Herpes infections of the skin were also not observed in controlled studies. With regard to the dreaded Eczema herpeticatum, even a clear protection could be achieved by effective therapy with dupilumab (OR 0.34), the same applies to bacterial skin infections (OR 0.54) [39, 40]. 

Fortunately, the antibody was also approved in 2019 for the treatment of allergic bronchial asthma, which often occurs together with severe neurodermatitis, so that in this case, two atopic diseases can now be treated with one antibody. With chronic rhinosinusitis with nasal polyps (see below), dupilumab was recently approved for another disease that often occurs together with atopic dermatitis. 

Hardly any other disease is currently so much in the focus of ongoing clinical studies with innovative drugs as atopic dermatitis. In the last 1.5 years alone, phase 2 studies with 6 further monoclonal antibodies were published as full papers [summarized under 41]. The most advanced clinical developments are the antibodies tralokinumab (anti-IL-13) and nemolizumab (anti-IL-31R), which have been shown to be effective in both eczema severity and subjective symptoms, especially pruritus. Lebrikizumab, another anti-IL-13 antibody, also showed convincing efficacy in a recently published phase 2 study, while fezakinumab (anti-IL-22), etokimab (anti-IL-33), and tezelumab (anti-TSLP) have only been the subject of smaller proof-of-concept studies for the indication atopic dermatitis [summarized in 41]. 

## Biologics for the therapy of chronic rhinosinusitis with nasal polyps 

The prevalence of chronic rhinosinusitis (CRS) is ~ 10 – 15% of the population in developed countries, which means a significant cost to health systems and economies [42, 43]. While the current phenotype classification is based on endoscopic examination of the nasal cavity or imaging techniques and divides CRSs into chronic rhinosinusitis with nasal polyps (CRSwNP) and chronic rhinosinusitis without nasal polyps (CRSsNP) [42, 44, 45], the focus of interest is increasingly shifting to the causative inflammatory pathomechanisms, according to which an endotype classification could be undertaken as soon as there is an internationally accepted consensus, and easy-to-identify and reliable biomarkers are developed [46]. 

Several studies have investigated the anti-IgE-antibody omalizumab in CRSwNP [47, 48, 49, 50]. A significant reduction of the nasal polyp score was shown in a randomized, double-blind placebo-controlled (DBPC) study in patients with CRSwNP and comorbid asthma [51]. Here, omalizumab therapy showed an effect on polyp scores comparable to a 3-week oral steroid treatment. 

A phase 2 study investigated the effect of omalizumab on CT morphological shading in the anterior ethmoid bone and maxillary sinus (polyp CT score) [52]. Two parallel double-blind, placebo-controlled phase 3 studies with omalizumab in CRSwNP (POLYP 1 and POLYP 2) investigated efficacy and tolerability in a large number of patients [53]. Compared to placebo, omalizumab showed statistically significant reductions in nasal polyp scores, nasal obstruction and other symptoms of CRSwNP. 

Omalizumab has been approved in Germany in 2020 as an adjunct therapy to intranasal corticosteroids (INCS) for the treatment of adults with severe CRSwNP in whom therapy with INCS does not provide adequate disease control. 

Two different strategies are available to block the IL-5-mediated inflammatory response: elimination of circulating IL-5 and blockade of the IL-5 receptor (IL-5R) on eosinophils and basophils [54, 55, 56]. 

In the treatment of steroid-refractory CRSwNP with mepolizumab, a significant improvement of polyp scores in CT and endoscopy and an improvement of olfactory function could be demonstrated even in the long-term effect 9 months after end of therapy [57]. A further study with mepolizumab to avoid the need for surgical sinus surgery using mepolizumab vs. placebo is currently still pending [58], as is the publication of the results of the pivotal phase 3 study [59]. 

The IL-5 antibody reslizumab has been tested in several placebo-controlled studies in asthma patients with comorbid nasal polyps and has been shown to improve quality of life [60, 61]. Also for the sole indication CRSwNP, promising results were obtained [62] with regard to polyp scores in CT of the paranasal sinuses and symptoms. 

Anti-IL5- and anti-IL-5R biologics such as benralizumab and TPI ASM8 [63] have not been used in nasal polyposis, but a DBPC phase 3 study to evaluate benralizumab in patients with CRSwNP is currently being completed (OSTRO study) [64]. 

Studies with anti-IL-4/anti-IL-13 antibodies aim to reduce pro-inflammatory markers of the Th2-mediated inflammatory response. The receptors of both cytokines share a common subunit (IL-4Rα), therefore blocking this subunit and thus both cytokines is a promising option [65, 66, 67]. In the indication CRSwNP, the monoclonal anti-IL-4Rα antibody dupilumab was evaluated in a DBPC phase 2 study over a treatment period of 4 months with significant improvements under dupilumab therapy for the primary endpoint of endoscopic polyp score [68]. 

In two phase 3 clinical trials (SINUS-24 and SINUS-52) with large patient numbers, dupilumab treatment in severe CRSwNP resulted in a statistically significant reduction in polyp size, reduction of shadows in the CT of the paranasal sinuses and improvement of disease symptoms [69]. 

Dupilumab has been approved in Germany since 2019 as an add-on therapy with intranasal corticosteroids for the treatment of adults with severe CRSwNP that cannot be adequately controlled with systemic corticosteroids and/or surgery. 

## Biologics for the treatment of hereditary angioedema 

In hereditary angioedema (HAE), recurrent edema of the skin and mucous membranes occurs in attacks. The prevalence of HAE is ~ 1 in 50,000 [70]. The cause of autosomal-dominantly inherited HAE type 1 and type 2 is a genetic defect in chromosome 11 that leads to a deficiency or malfunction of the C1 inhibitor (C1-INH). Other C1-INH-independent types are caused by mutations of factor XII, plasminogen, or angiopoetin. In addition, there appear to be other, as yet unidentified mutations [70, 71]. The kallikrein-kinin system, C1-INH, and bradykinin play an important pathophysiological role. In addition to the administration of C1-INH preparations, drugs that act on the bradykinin system are now also used therapeutically in HAE [71]. Depending on the frequency and severity of the attacks, a distinction must be made in the care of HAE patients between acute treatment, short-term and long-term prophylaxis [71]. 

In early 2019, lanadelumab, a new drug for the long-term prevention of HAE, became available on the German market. Lanadelumab is a recombinant, fully humanized immunoglobulin G1-kappa light chain monoclonal antibody [72]. Subcutaneous administration is performed regularly every 14 days; an extension of the dose intervals is possible. Due to the highly potent and specific inhibition of plasma kallikrein, lanadelumab leads to a sustained inhibition of plasma kallikrein activity [72]. The efficacy and safety of lanadelumab for the long-term prevention of HAE attacks in patients with confirmed C1-esterase-inhibitor-induced HAE aged 12 years and older has been investigated in several studies. The studies showed a significant reduction in the attack rate of HAE in the actively treated groups compared to placebo and an increase in the percentage of patients without attacks. Treatment with lanadelumab was generally safe and well tolerated, with local reactions at the injection site being the most common treatment-related adverse events [73, 74, 75]. Garadacimab (CSL132, NCT03712228), a human IgG4 antibody for subcutaneous administration, is another biological agent in clinical trials for use in HAE. Garadacimab binds and inhibits activated factor XIIa, thereby inhibiting bradykinin formation and preventing the development of HAE attacks. In a phase 2 study in 32 patients with C1-INH-dependent HAE, a significant reduction in the number and severity of attacks was achieved in the three actively treated groups compared to placebo. Mild local reactions were observed in 12.5% [76]. 

## Biologics for the treatment of food allergy 

IgE-mediated food allergy is a potentially life-threatening disease for the treatment of which there is no approved biological agent yet. Based on the pathophysiology of the disease or the mechanism of action of omalizumab, it is believed that this substance is effective in IgE-mediated food allergy. Accordingly, numerous case series and individual controlled prospective studies with a limited number of cases, mostly in children, show that omalizumab is effective as monotherapy or in combination with oral immunotherapy. 

Monotherapy with anti-IgE can raise the tolerance threshold of the food allergen in question. Most cases have been reported with peanuts [77, 78], but other foods such as cow’s milk [79] and hen’s egg have also increased the maximum tolerated dose after several months of treatment with anti-IgE [80]. A recent study investigated the efficacy of anti-IgE treatment in children who were allergic to several foods [81]. The results of the study show that the group treated with omalizumab was significantly more likely to reach 2 g protein in more than 2 of the food allergies compared to placebo. 

These data show that even in patients with several food allergies, omalizumab can improve the efficacy of oral immunotherapy. 

Another therapeutic approach is to reduce the rate of side effects or to enable a faster dosage of the food allergen by administering anti-IgE during oral immunotherapy. Again, the study results show efficacy of omalizumab in peanut-allergic children [82] as well as in patients with multiple food allergies compared to placebo-treated patients [83, 84]. 

In summary, the data available to date are promising with very good tolerability, but there are still open questions such as the optimal dose and the treatment regimen. 

The next-generation anti-IgE ligelizumab is of future interest. It has already shown very good results in the treatment of chronic spontaneous urticaria. Due to its biological properties, it will be an interesting new molecule for the treatment of food allergy in the future. 

Dupilumab also has potential for clinical use in food allergy due to its ability to downregulate the IgE response during treatment. First studies have started, and the results are eagerly awaited. Ultimately, the great hope is that safe and effective new biologically active substances for food allergy will be available for therapy to effectively treat patients with potentially life-threatening diseases [85]. 

## Hypersensitivity reactions to biologicals 

Among the adverse effects of biologicals, the “cytokine storm” and the IgE- and non-IgE-mediated anaphylactic/anaphylactoid reaction are the most feared. Pichler [86] classified the side effects of biologicals into five types (alpha, beta, gamma, delta, epsilon) and thus made the sometimes very unusual adverse events clinically more comprehensible in terms of diagnostics, therapy, and prevention. Only the alpha- and beta-type reactions will be discussed in more detail here. 

Type alpha reactions are based on a direct effect on immune stimulation by cytokine release. They are direct substance-dependent, dose-dependent effects right from the first application. They are among the most frequent reactions and decrease again in the course of therapy. 

Type beta reactions are hypersensitivity reactions, including allergies of type I – IV, i.e., immune reactions to the therapeutic protein. They are unpredictable, do not occur during first application apart from the anaphylaxis due to cetuximab (see below), and are rather independent of the dose. 

Both types of reaction can be life-threatening and may produce symptoms that meet the anaphylaxis criteria. To date, the non-IgE-mediated response and the “cytokine storm” are not fully pathophysiologically/mechanistically understood, making classification difficult [87]. These reactions are important not only because they can be life-threatening, but also because their symptomatology generally leads to the termination of the triggering biological therapy, which is very detrimental to patients with regard to their underlying disease. Therefore, the goal must be to understand these reactions fundamentally and to diagnose them more reliably in order to derive a better management of these severe side effects in favor of a safe and efficient therapy of the underlying disease. 

On the other hand, the therapy of the cytokine release syndrome is different from that of anaphylaxis! 

A temporary discontinuation of the biological therapy and a new start with slower infusion rate as well as premedication with antihistamines and glucocorticoids can be helpful. In case of anaphylaxis, premedication does not help causally. Furthermore, the risk of subsequent anaphylaxis is high [87]! 

However, a comprehensive review of databases and scientific literature has shown that, on the one hand, the nomenclature of hypersensitivity reactions to biologicals is not harmonized, so that data on the prevalence and incidence of “real” allergic and anaphylactic reactions to the various biologicals cannot be reliably collected [88, 89]. Furthermore, the symptomatology of anaphylaxis may vary between different biologicals [87]. Only the careful characterization of patients with such reactions in registries will be able to remedy this situation. 

The immunogenicity of biologics depends mainly on the degree of their humanization: Chimeric monoclonal antibodies, such as cetuximab and infliximab, which are produced in a mouse hybridoma cell line (SP2/0), have immunogenic murine components. The now best-known IgE epitope is the disaccharide alphaGAL, which was discovered by anaphylaxis due to cetuximab after initial application with detection of pre-existing IgE antibodies against this structure and is also responsible for the delayed anaphylaxis due to mammalian meat. The main sensitization pathway is now considered to be tick bites, in the USA the species *Amblyomma americanum* is responsible [90]. Another association with anti-alphaGal IgE has only been described for infliximab [91]. There are reports of IgE antibody detection against biologicals that triggered anaphylactic reactions (summarized by Joshi and Khan, 2019 [87]). A group of Italian authors showed that patients with IgE against the relevant biological in serum and/or skin tests with this biological reacted more rapidly (3^rd^ dose) and more severely [92, 93]. To date, there is no routine procedure available for this. (The detection of antibodies directed against biologicals in sera of treated patients is routinely performed only for the detection of neutralizing antibodies, which are mostly of the IgG type). 

However, the fact that the degree of humanization of biologicals reduces their immunogenicity does not exclude the formation of anti-drug antibodies (ADA) against non-self sequences of fully human therapeutic antibodies [86]. 

Anti-infliximab IgG is detectable in sera of patients with anaphylaxis due to infliximab during infusions [92, 94] as well as IgM, but the clinical relevance of IgM remained unclear. Matucci et al. [93] and Hwang et al. [95] described the possibility of using anti-infliximab antibody detection to assess the risk of developing a reaction. 

For patients living in endemic areas with a high prevalence of alphaGAL sensitization, the determination of IgE antibodies against alphaGAL prior to cetuximab administration is useful [90, 96]. For this purpose, alphaGAL is available in the form of bovine thyroglobulin in the ImmunoCAP (ThermoFisher Scientific/Phadia, Freiburg, Germany). 

However, additives such as polysorbate, mannitol, albumin, latex, trometamol, and papain [89, 97] can also cause allergic reactions to biologicals and should be included in the allergological investigation. 

**Figure 1.**
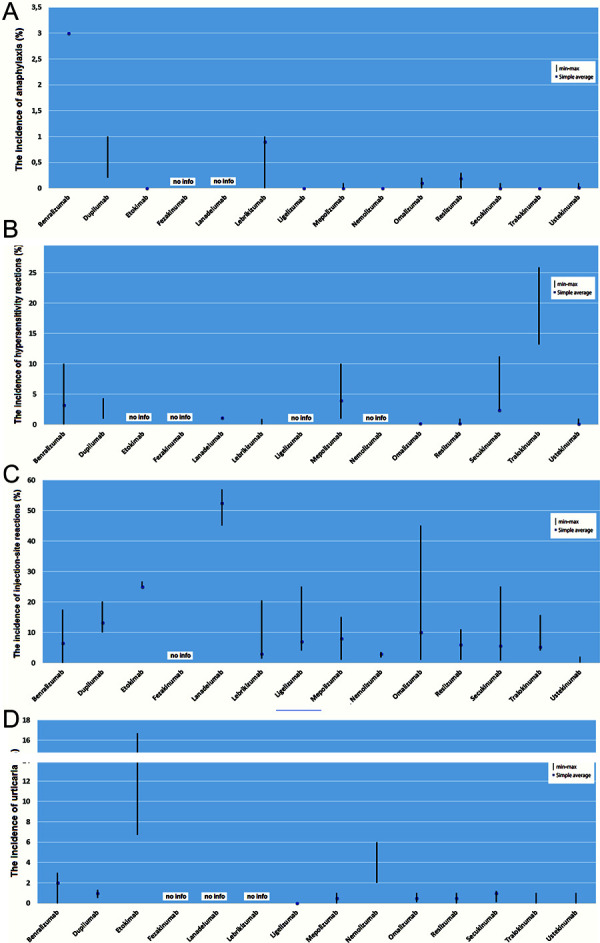
Incidences of different hypersensitivity reactions to biologics.

For the biologicals listed in this overview under the various indications for the therapy of atopic diseases, the frequency of hypersensitivity reactions is shown in Table 1 and Figure 1 according to the research on data bases. Recently, the case of a serum disease-like reaction to dupilumab was described [98]. 

The diagnostic measures to detect a rather rare IgE-mediated adverse reaction are, in addition to the medical history (occurrence and progression of the reaction in the course of therapy, relative independence from the administered dose, method of application, duration of therapy and therapy pause, if applicable life in an alphaGAL sensitization endemic area, mammalian meat allergy), the prick and intradermal test with the suspected biological, which, however, corresponds to an off-label use about which the patient should have been informed and given written consent. In general, allergy diagnostics should be performed within 4 – 6 weeks after the event to be meaningful [89]. 

According to our own data, antibody-based diagnostics of biological hypersensitivity reactions should be expanded in order to detect pre-existing antibodies before starting a biological therapy or to detect their development during the course of therapy [99, 100] and to exclude possible allergen or epitope similarities between the biological that causes undesirable immunological side effects and the one to be switched to. This way, the change to a safe and efficient biological therapy can be largely ensured in the future. 

## Treatment with biologics and vaccinations 

Biologics therapy massively interferes with immune regulation. The question always arises whether this has an effect on the defense against infectious agents, i.e., bacteria, viruses, fungi, and parasites. Specifically, the question is whether the respective biological therapy increases the readiness for infection. Parallel to this, vaccination programs are being carried out very successfully against many pathogens today. Here the question arises whether patients under biological therapy also benefit from vaccinations (inactivated or attenuated vaccines), or whether – especially through the administration of attenuated vaccines – there is an increased risk of a flare-up or development of a corresponding infectious disease under biological therapy. 

Biologics therapy has been in use for the longest time and is most widely used in the context of rheumatoid arthritis. Biologic therapy, especially with TNF antagonists, has revolutionized the treatment of rheumatoid arthritis. This is why most experience in this field is available in terms of infection risks and vaccination responses. Anti-TNF therapy has been described as having increased rates of infection with Varicella zoster virus (this is a reactivation), chronic hepatitis B virus infection and CMV infection. For this reason, the immune status with regard to these pathogens (and also other infections) should be examined before therapy with TNF antagonists. There is a pragmatic suggestion for this in the literature [101], and it can also be transferred to a therapy with biologicals for allergy and asthma (Table 2). Therefore, it is recommended to check the immune status with regard to these important viruses (and bacteria) before starting a biological therapy and, if necessary, to refresh vaccinations or make up for missing vaccinations before initiating a biological therapy. 

In the case of bacterial diseases, the focus is on tuberculosis, especially with regard to biological therapy for autoimmune diseases. Here it could be shown that therapy with TNF antagonists leads to an increased risk of a mycobacterial infection flaring up. On the other hand, there is no increased risk with a biological therapy directed against CD20, IL-6 receptor, IL-12/IL-23, and CD80/CD86 [102]. 

Vaccination data are also available for some biologics that are now approved for allergy and asthma therapy. With regard to vaccinations against bacterial pathogens, the tetanus vaccination is worth mentioning. Here, it could be shown for the therapy with dupilumab (inhibition of the IL-4 and IL-13 signaling pathways) that there is no impairment of the development of a tetanus titer response [103] or a bactericidal response detectable in serum. It can therefore be concluded that patients treated with this biological agent can also receive inactivated or attenuated vaccines at the same time. With regard to viral infections, influenza vaccination is of particular importance, especially since patients with asthma have an increased risk of a (severe) influenza infection. In this context, therapy with a monoclonal anti-IL-5 receptor antibody (benralizumab) has been shown to not limit the antibody response under seasonal influenza vaccination in adolescents and young adults with moderate to severe asthma [104]. 

However, it has to be emphasized that it is not possible to draw conclusions from one vaccine to the other in principle, as vaccinations against different pathogen classes (viruses and bacteria) also activate or use different immunological strategies when using drugs with completely different configurations (e.g., attenuated and inactivated vaccines, significance of the added adjuvant). Therefore, there is still a considerable need for studies (regarding the number of vaccinated patients under biological therapy, the use of different vaccines against different pathogens and regarding the long-term course). Only then can a conclusive and comprehensive picture be drawn on this important topic. 

## Biologics in pregnancy and childhood 

Most scientific publications and studies on biologicals in pregnancy refer to autoimmune or inflammatory chronic diseases, such as rheumatoid arthritis, lupus erythematosus, or psoriasis vulgaris. Active autoimmune diseases involve an increased risk of adverse maternal and fetal events such as pre-eclampsia, miscarriage, intrauterine growth disorders, preterm birth, or low birth weight [105]. For example, the treatment goal for rheumatoid arthritis is to have little or no pre-conception activity, as negative effects of steroids and non-steroidal anti-inflammatory drugs must be considered [106]. Experiences from case reports and registry data with TNF antagonists, which have been approved for many years for the treatment of rheumatological diseases and psoriasis vulgaris, have so far shown no evidence of an increased number of spontaneous abortions or malformations [107]. As a result, the use of TNF inhibitors such as infliximab, adalimumab, and etanercept is recommended in pregnancy up to week 20. A newer antibody, certolizumab, has been shown to be safe for the entire pregnancy [108, 109, 110]. There is limited data on the newer biologics, such as ustekinumab, secukinumab, ixekizumab, and brodalumab, and their use in pregnancy is currently not recommended, mainly as a precaution [110]. Omalizumab, which has been approved and used for the longest time in allergology, was investigated in the “Expect Study” [111]. In this study, 250 women with asthma who received omalizumab during pregnancy were examined. The data show no evidence of an increased risk of congenital malformations. However, there are still no recommendations in the international guidelines. The other antibodies used in allergology, such as benralizumab, reslizumab, and dupilumab, are not recommended for use in pregnancy due to lack of data. However, due to their mechanism of action, no increased risk can be assumed, and on the other hand, unstable chronic diseases have to be considered in the context of increased use of, for example, oral corticosteroids. In the future, further register-based data or case-control studies will be required to establish the evidence for the safe use of biologicals in pregnancy also in allergology. 

## Biologics in patients with uncertain SARS-CoV-2 infection status 

The pandemic SARS-CoV-2 infection, which is still being researched pathophysiologically, has caused uncertainty for potential risk groups of patients regarding the therapy regimen of chronic inflammatory and oncological diseases, i.e., especially those diseases that are treated immunosuppressively and/or with biologicals. This concerns acute care as well as the treatment of chronically ill patients. Up to now, only little is known about the immune response after SARS-CoV-2 infection and could be changed favorably or unfavorably by a therapy with monoclonal antibodies. The current study situation [cited in 112] does not provide evidence of an increased risk of allergic patients for a more severe COVID-19 disease course, but reliable data are lacking. Numerous patients receive biologicals that inhibit type 2 immune responses via different mechanisms. A selective literature search was carried out in Pubmed, Livivo, and on the World Wide Web for the past 10 years (period 05/2010 – 04/2020). The current German-language publications not included in this search were analyzed and a position paper with recommendations for treatment with biologicals in patients with allergic and atopy-associated diseases in the COVID-19 pandemic was compiled [112]. Until study data are available, all patients under therapy with a biological agent directed against type 2 immune reactions who are suffering from COVID-19 should be registered and well characterized. In this way, the basis for experience- and data-based instructions for action can be created. The position paper recommends the continuation of therapy of bronchial asthma, atopic dermatitis, chronic rhinosinusitis with nasal polyps and spontaneous urticaria with biologicals during the SARS-CoV-2 pandemic in patients without suspected or proven SARS-CoV-2 infection [112]. The aim is to optimally control difficult-to-control allergic and atopic diseases through appropriate medication-as-needed and add-on therapy and to avoid the need for systemic glucocorticosteroids. Since there is no reliable knowledge about the effect of the biologicals on the immunological situation of SARS-CoV-2 infection and COVID-19, the therapy should be decided on individually together with the patient after a risk-benefit analysis in case of justified suspicion or proof of an infection with SARS-CoV-2. 

## New biologics – this could be what we can expect in the next few years 

Biologics have also revolutionized therapy in the field of allergic diseases in recent years. Currently, several promising biologicals for different indications are being studied in clinical trials. An excerpt from the diverse areas of application was examined more closely by Prof. Bernhard Homey (atopic dermatitis; AD), Dr. Sebastian Reuter (bronchial asthma), and Dr. Mandy Cuevas (nasal polyps) at the symposium “New Biologics in Studies” organized by the DGAKI Junior Members in cooperation with the still young working group Biologics and New Pharmaceuticals of the DGAKI at the 14^th^ German Allergy Congress in Hannover, Germany. In the following, the biologics currently being studied for these indications will be summarized based on the presentations. 

Following the successful approval of the anti-IL-4 receptor antibody dupilumab for adults and children over 12 years of age with moderate to severe AD [113, 114], approval could soon be extended to children under 12 years of age, as a recently completed study by Cork et al. suggests [113]. 

Two other biologics are likely to become available soon for the treatment of moderate to severe AD, the anti-IL-13 antibody tralokinumab and the JAK inhibitor baricitinib. Phase 3 trials of both tralokinumab and baricitinib have reached their primary endpoints [115, 116], and the manufacturer of tralokinumab reports that a marketing authorization application has already been approved by the European Medicines Agency. As a result, tralokinumab will soon be available for the treatment of AD, and baricitinib may be next. 

There are, however, other cytokine- or receptor-targeted biologics currently being evaluated in clinical trials, including the anti-IL-13 antibody lebrikizumab, whose phase 2 study results showed an early treatment response and a safe to acceptable risk profile [117]. Phase 3 studies are currently being conducted. In addition, IL-17 is also an interesting target in AD. The anti-IL-17a antibody secukinumab, which is already approved for psoriasis, is currently being evaluated in a phase 2 clinical trial for the treatment of AD. The anti-IL-22 antibody fezakinumab was also recently tested in a phase 2 study [118]. Response rates of patients with severe AD were better than those of patients with moderate AD. There was a significant superiority in response when compared to placebo with good tolerability [118, 119]. The monoclonal antibody nemolizumab is directed against IL-31RA. A phase 2 study over 52 weeks showed sustained efficacy and good tolerability [120]. Furthermore, the phase 2 study with the anti-IL-33 antibody etokimab has just been completed. Preliminary results made a promising impression [121]. However, it appears that the primary endpoint of the study could not be met. 

After some biologics (omalizumab, ustekinumab, MOR106) failed in the indication AD, there are currently some promising candidates that could become available for the treatment of AD in addition to dupilumab in the next few years. 

For many years, the use of biologics in nasal polyposis has been predominantly in patients with comorbidity to severe asthma or as off-label use. However, targeted registration studies are currently being conducted for various biologics in nasal polyposis [summarized in 122]. 

Patients with CRSwNP have a significant reduction in quality of life, sleep quality, and daily productivity due to nasal obstruction, anterior and posterior secretion, and associated facial pain and olfactory disorders. The established treatment options so far are drug therapy (steroid-containing nasal sprays) and surgical measures (surgical removal of polyps). However, the risk of recurrence is high, and therefore not every patient can be treated satisfactorily. In CRSwNP, besides the IgE-mediated allergic reaction, the importance of Th2 cells and their mediators in the development and maintenance of the disease is well known. For this reason, approaches of targeted therapy with biologicals that inhibit this signaling pathway have been increasingly pursued in recent years. Phase 3 studies with the anti-IgE antibody omalizumab, the anti-IL-5 antibodies mepolizumab and reslizumab, the anti-IL-5Rα antibody benralizumab and the anti-IL-4R antibody dupilumab show promising results [123]. 

With the European approval of dupilumab in autumn 2019 as an add-on therapy with intranasal glucocorticoids for the treatment of adults with severe CRSwNP, which cannot be adequately controlled by systemic glucocorticoids and/or surgery, a biological agent for the primary therapy of CRSwNP is available for the first time and is prescribable and reimbursable in Germany. 

Recent years have brought significant progress in the treatment of bronchial asthma. In particular, the more precise definition of clinical phenotypes and immunological endotypes allows a more targeted therapy of patients [124]. Beneficiaries of this are the previously therapy-refractory severe asthmatics with eosinophilia, the so-called type 2 high asthma [125]. With the antibodies against IL-5 (mepolizumab, reslizumab), against IL-5R (benralizumab), and against the alpha subunit of IL-4R (dupilumab), four candidates have been launched on the market that focus on this endotype [14, 126]. 

Another promising approach is the suppression of alarmins, such as IL-33 and TSLP, which are messengers of epithelial cells at the beginning of the inflammation cascade. The inhibition of these immunomodulators could already reduce or prevent the inflammatory reaction in its development [127]. An antibody against IL-33 (etokimab) was shown to improve FEV1 levels and eosinophilia in blood in a phase 2 study. The TSLP-neutralizing antibody tezepelumab showed a significant improvement in annual exacerbations in a phase 2 study. Another interesting result of the TSLP study was that not only asthmatics with type 2-high benefited from the new therapy, but also those with type 2-low, for whom no biologicals were previously available [128, 129]. 

Asthmatics with this endotype often show a neutrophilic inflammatory response and respond less well to corticosteroids. Type 2-low asthma is much less well understood than type 2-high asthma, but we do know that Th1 and Th17 cells and their mediators orchestrate the neutrophil inflammatory response [130]. First biologics that specifically target this endotype by suppressing the IL-17 and TNF target structures did not achieve the desired effects [131, 132, 133]. Preliminary results on an antibody against CXCR2 (AZD5069) are more promising. CXCR2 is a receptor on neutrophils whose blockade prevents activation by IL-8. In initial studies, the antibody showed good safety and reduced neutrophil numbers, but could not show a significant effect on exacerbation rates [134, 135]. 

Overall, we now have targeted therapeutics for the different phenotypes of atopic diseases. 

In view of the rapidly developing study situation for possible new marketing authorizations, care must be taken with regard to the observation and documentation of side effects. For this purpose, the registration of patients treated with biologicals in registries is a methodically sound way. But also the research and development of suitable diagnostic methods for the registration of immunologically caused side effects or the registration of the “theratype”, i.e., the differentiation of potential therapy responders from non-responders, is certainly of higher importance than commonly assumed so far. 

## Conflict of interest 

CT, MRE, UJ, AG, HB, JP, KCB have no conflict of interest. 

RT received research support from Sanofi-Genzyme and Hautnetz Leipzig/Westsachsen e.V. as well as fees for lectures and consulting from ALK-Abello, Takeda, Novartis, Sanofi-Genzyme, Abbvie and support for congress visits from Takeda. 

LK reports on grants and/or personal fees from Allergopharma, MEDA / Mylan, HAL Allergy, ALK Abelló, LETI Pharma, Stallergenes, Quintiles, Sanofi, ASIT Biotech, Lofarma, Allergy Therapist, AstraZeneca, GSK, Inmunotk and Cassela med outside of the submitted paper; and memberships: AeDA, DGHNO, German Academy for Allergology and Clinical Immunology, ENT-BV, GPA, EAACI. 

MW reports on support for consultations, lectures and other scientific activities by ALK-Abelló Arzneimittel GmbH, Abbvie, Eli Lilly, Mylan Germany GmbH, Bencard Allergie GmbH, Novartis AG, Biotest AG, Sanofi-Aventis Deutschland GmbH, HAL Allergie GmbH, DBV Technologies S.A, Aimmune Therapeutics UK Limited, Regeneron Pharmaceuticals, Inc, Stallergenes GmbH. 

TZ reports support for consultations, lectures, and other scientific activities by AstraZeneca, AbbVie, ALK, Almirall, Astellas, Bayer Health Care, Bencard, Berlin Chemie, FAES, HAL, Henkel, Kryolan, Leti, Lofarma, L’Oreal, Meda, Menarini, Merck, MSD, Novartis, Pfizer, Sanofi, Sanoflore, Stallergenes, Takeda, Teva, UCB as well as responsible collaboration in the following organizations: Committee member, WHO initiative “Allergic Rhinitis and its Impact on Asthma” (ARIA), Member of the Board, German Society for Allergy and Clinical Immunology (DGAKI), Head, European Centre for Allergy Research Foundation (ECARF), Secretary General, Global Allergy and Asthma European Network (GA^2^LEN), Member, Committee on Allergy Diagnosis and Molecular Allergology, World Allergy Organization (WAO). 

TW reports support for consultations, lectures, and other scientific activities by AbbVie, ALK Abello, Almirall, Astellas, Bencard, Galderma, Janssen/JNJ, Leo Pharma, Leti, Lilly, Novartis, Pfizer, Regeneron/Sanofi, Stallergen. 

MWa has received fees for consulting, presentations or research support from the following companies within the past 3 years: ALK-Abelló, Allergopharma, AstraZeneca, Bencard Allergy, Genzyme, HAL Allergy, Infectopharm, LETI Pharma, MEDA Pharma, Novartis, Sanofi Aventis, Stallergenes, Teva – outside of the present work 

HR: Co-Founder STERNA Biologicals and SECARNA Pharmaceuticals. 

**Table 1.** Published reports on the frequencies of hypersensitivity reactions to biologics.

Biologic	Target	Author	Year	HSR	IR	ISR	Urticaria	Anaphylaxis
Omalizumab	IgE	Cox et al. [136] Di Bona et al. [137] FDA [138]^a ^ FDA [138]^b^ EMA [139]	2007 2017 2019 2019 2019	< 0.2 – – – –		– 3.4 12.0 – 45.0 0.6 – 2.7 1.0 – 10.0	– 1.0 0.2 – 0.1 – 1.0	0.09 0 0.1 – 0.2
Ligelizumab	Cε3 domain of IgE	Gauvreau et al. [140] Maurer et al. [141]	2016 2019	– –		12.5–25.0 4.0–7.0	0 –	0 0
Mepolizumab	IL-5	Pavord et al. [142] Lugogo et al. [143] Khatri et al. [144] FDA [145] EMA [146] Chapman et al. [147]	2012 2016 2019 2019 2019 2019	≤ 1.0 < 1.0 2.0 1.0 – 4.0 1.0 – 10.0 < 1.0	5.0 – 12.0 < 1.0 – – 1.0–10.0 –	– 3.0 12.0 8.0 – 15.0 1.0 – 10.0 3.0	– – – – – < 1.0	0 0 0 – 0.1 – 0.01 0
Reslizumab	IL-5	Castro et al. [60] Murphy et al. [148] FDA [149] EMA [150] Bernstein et al. [151]	2015 2017 2019 2019 2020	– < 1.0 – 0.19 0	– < 1.0 – 0.19 –	1.0 – 2.0 < 1.0 – – 6.0 – 11.0	– < 1.0 – – –	< 1.0 0 0.3 0.19 –
Benralizumab	IL-5Rα	Castro et al. [152] Park et al. [153] Liu et al. [154] FDA [155] EMA [156] Bourdin et al. [157]	2014 2019 2019 2019 2019 2019	– – – 3.0 1.0 – 10.0 0 – 3.2		16.0 0 2.6 – 17.5 2.2 2.2 3.2 – 6.5	– 0 – 2.0 – 3.0 – –	– – – 3.0 ? –
Dupilumab	IL-4Rα	Ou et al. [158] EMA [159] FDA [160]	2018 2019 2020	– 3.0 – 4.3 < 1.0		13.2 16.0 – 20.1 10.0	– 0.5 – 1.3 < 1.0	– 0.2 < 1.0
Lanadelumab	Plasma kallikrein	FDA [161] EMA [162]	2018 2020	1.0 1.2		45 – 57.0 52.4	– –	– –
Lebrikizumab	IL-13	Hanania et al. [163] Hanania et al. [164] Simpson et al. [117] Korenblat et al. [165]	2015 2016 2018 2018	0 – 0.9 – – –		11.1 – 20.5 6 – 10.0 1.3 2.9	– – – –	0 – 0.9 < 1.0 0 1.0
Tralokinumab	IL-13	Wollenberg et al. [166] Panettieri et al. [167] Busse et al. [168] Carlsson et al. [169]	2019 2018 2019 2019	– – –13.2 – 25.9		5.2 4.0 – 5.4 15.7 –	– – – < 1.0	– 0 0 0
Secukinumab	IL-17A	EMA [170] Blauvelt [171] Deodhar et al. [172] FDA [173] EMA [174] Grace et al. [175]	2015 2016 2019 2020 2020 2020	6.5–11.2 – 2.4 – – –		5.6 0.7 0.8 – 1.3 – – 25.0	< 1.0 – – 0.6 – 1.2 0.1 – 1.0 –	0 – – – < 0.1 –
Fezakinumab	IL-22	–	–	–	–	–	–	–
Nemolizumab	IL-31Rα	Nemoto et al. [176] Kabashima et al. [120] Silverberg et al. [177] Ständer et al. [178]	2016 2018 2020 2020	– – – –	– – – –	– 2.0 1.8 – 3.5 3.0	– 2.0–6.0 – –	0 – – –
Etokimab	IL-33	Chen et al. [121] Chinthrajah et al. [179]	2019 2019	– –	– –	25.0 26.7	16.7 6.7	– 0
Ustekinumab	IL-12/IL-23	Ghosh et al. [180] FDA [181] EMA [182]	2019 2020 2020	< 1.0 0.08 0.1 – 1.0	0.1 – 0.1	– 1.0 – 2.0 0.1 – 1.0	< 1.0 < 1.0 –	0 0.1 0.01 – 0.1

^a^Results of clinical studies with asthma in FDA 2019 label; ^b^results of pooled Chronic Idiopathic Urticaria trials in FDA 2019 label. HSR = hypersensitivity reaction; IR = infusion reaction, substance-specific; ISR = injection-site-reaction.

**Table 2.** Laboratory tests before administration of immunosuppressive or immunomodulating drugs.

Virus	Test
Hepatitis B virus	– Anti-HBS quantified – HBs antigen, anti-HBs, and – Anti-HBc
Hepatitis C virus	(Anti-hepatitis C)
Hepatitis A virus	(Anti-HAV IgG)
Epstein-Barr virus	Anti-EBV
Cytomegalovirus	Anti-CMV IgG and IgM
Herpes virus	Anti-HSV q and 2: IgG and IgM
Varizella-Zoster virus	Anti-VZ IgG
Syphilis	VDRL or TPPA

## References

[1] van Buul AR Taube C Treatment of severe asthma: entering the era of targeted therapy. Expert Opin Biol Ther. 2015; 15: 1713–1725. 26331583 10.1517/14712598.2015.1084283

[2] Hekking PPW Wener RR Amelink M Zwinderman AH Bouvy ML Bel EH The prevalence of severe refractory asthma. J Allergy Clin Immunol. 2015; 135: 896–902. 25441637 10.1016/j.jaci.2014.08.042

[3] Taube C Bramlage P Hofer A Anderson D Prevalence of oral corticosteroid use in the German severe asthma population. ERJ Open Res. 2019; 5: 00092-2019902 31687373 10.1183/23120541.00092-2019 PMC6819991

[4] Haasler I Taube C [Biologicals in the treatment of bronchial asthma]. Pneumologie. 2017; 71: 684–698. 29017221 10.1055/s-0043-102773

[5] Chung KF Wenzel SE Brozek JL Bush A Castro M Sterk PJ Adcock IM Bateman ED Bel EH Bleecker ER Boulet LP Brightling C Chanez P Dahlen SE Djukanovic R Frey U Gaga M Gibson P Hamid Q Jajour NN International ERS/ATS guidelines on definition, evaluation and treatment of severe asthma. Eur Respir J. 2014; 43: 343–373. 24337046 10.1183/09031936.00202013

[6] Buhl R Bals R Baur X Berdel D Criée CP Gappa M Gillissen A Greulich T Haidl P Hamelmann E Kardos P Kenn K Klimek L Korn S Lommatzsch M Magnussen H Nicolai T Nowak D Pfaar O Rabe KF [Guideline for the diagnosis and treatment of asthma – Guideline of the German Respiratory Society and the German Atemwegsliga in Cooperation with the Paediatric Respiratory Society and the Austrian Society of Pneumology]. Pneumologie. 2017; 71: e3 30406626 10.1055/a-0790-0021

[7] Sweeney J Patterson CC Menzies-Gow A Niven RM Mansur AH Bucknall C Chaudhuri R Price D Brightling CE Heaney LG Comorbidity in severe asthma requiring systemic corticosteroid therapy: cross-sectional data from the Optimum Patient Care Research Database and the British Thoracic Difficult Asthma Registry. Thorax. 2016; 71: 339–346. 26819354 10.1136/thoraxjnl-2015-207630

[8] Braunstahl GJ Chlumský J Peachey G Chen CW Reduction in oral corticosteroid use in patients receiving omalizumab for allergic asthma in the real-world setting. Allergy Asthma Clin Immunol. 2013; 9: 47 24305549 10.1186/1710-1492-9-47 PMC3879326

[9] Humbert M Taillé C Mala L Le Gros V Just J Molimard M Omalizumab effectiveness in patients with severe allergic asthma according to blood eosinophil count: the STELLAIR study. Eur Respir J. 2018; 51: 170523 10.1183/13993003.02523-2017 PMC6383600 29545284

[10] Ortega HG Liu MC Pavord ID Brusselle GG FitzGerald JM Chetta A Humbert M Katz LE Keene ON Yancey SW Chanez P Mepolizumab treatment in patients with severe eosinophilic asthma. N Engl J Med. 2014; 371: 1198–1207. 25199059 10.1056/NEJMoa1403290

[11] FitzGerald JM Bleecker ER Nair P Korn S Ohta K Lommatzsch M Ferguson GT Busse WW Barker P Sproule S Gilmartin G Werkström V Aurivillius M Goldman M Benralizumab, an anti-interleukin-5 receptor α monoclonal antibody, as add-on treatment for patients with severe, uncontrolled, eosinophilic asthma (CALIMA): a randomised, double-blind, placebo-controlled phase 3 trial. Lancet. 2016; 388: 2128–2141. 27609406 10.1016/S0140-6736(16)31322-8

[12] FitzGerald JM Bleecker ER Menzies-Gow A Zangrilli JG Hirsch I Metcalfe P Newbold P Goldman M Predictors of enhanced response with benralizumab for patients with severe asthma: pooled analysis of the SIROCCO and CALIMA studies. Lancet Respir Med. 2018; 6: 51–64. 28919200 10.1016/S2213-2600(17)30344-2

[13] Nair P Wenzel S Rabe KF Bourdin A Lugogo NL Kuna P Barker P Sproule S Ponnarambil S Goldman M Oral glucocorticoid-sparing effect of benralizumab in severe asthma. N Engl J Med. 2017; 376: 2448–2458. 28530840 10.1056/NEJMoa1703501

[14] Castro M Corren J Pavord ID Maspero J Wenzel S Rabe KF Busse WW Ford L Sher L FitzGerald JM Katelaris C Tohda Y Zhang B Staudinger H Pirozzi G Amin N Ruddy M Akinlade B Khan A Chao J Dupilumab efficacy and safety in moderate-to-severe uncontrolled asthma. N Engl J Med. 2018; 378: 2486–2496. 29782217 10.1056/NEJMoa1804092

[15] Bergmann KC Maurer M Church MK Zuberbier T Anaphylaxis to mepolizumab and omalizumab in a single patient: Is polysorbate the culprit? J Investig Allergol Clin Immunol. 2020; 30: 285–287. 10.18176/jiaci.0492 32723701

[16] Bölke G Church M Bergmann KC Comparison of extended intervals and dose reduction of omalizumab for asthma control. Allergo J Int. 2019; 28: 1–4.

[17] Dressler C Rosumeck S Werner RN Magerl M Metz M Maurer M Nast A Zuberbier T Executive summary of the methods report for “The EAACI/GA2 LEN/EDF/WAO Guideline for the Definition, Classification, Diagnosis and Management of Urticaria. The 2017 Revision and Update”. Allergy. 2018; 73: 1145–1146. 29336489 10.1111/all.13414

[18] Kaplan A Ledford D Ashby M Canvin J Zazzali JL Conner E Veith J Kamath N Staubach P Jakob T Stirling RG Kuna P Berger W Maurer M Rosén K Omalizumab in patients with symptomatic chronic idiopathic/spontaneous urticaria despite standard combination therapy. J Allergy Clin Immunol. 2013; 132: 101–109. 23810097 10.1016/j.jaci.2013.05.013

[19] Saini SS Bindslev-Jensen C Maurer M Grob JJ Bülbül Baskan E Bradley MS Canvin J Rahmaoui A Georgiou P Alpan O Spector S Rosén K Efficacy and safety of omalizumab in patients with chronic idiopathic/spontaneous urticaria who remain symptomatic on H1-antihistamines: a randomized, placebo-controlled study. J Invest Dermatol. 2015; 135: 925 10.1038/jid.2014.512 25501032

[20] Maurer M Rosén K Hsieh HJ Saini S Grattan C Gimenéz-Arnau A Agarwal S Doyle R Canvin J Kaplan A Casale T Omalizumab for the treatment of chronic idiopathic or spontaneous urticaria. N Engl J Med. 2013; 368: 924–935. 23432142 10.1056/NEJMoa1215372

[21] Vadasz Z Tal Y Rotem M Shichter-Confino V Mahlab-Guri K Graif Y Kessel A Agmon-Levin N Maoz-Segal R Kivity S Benor S Lachover-Roth I Zeldin Y Stein M Toker O Hassoun G Bezalel-Rosenberg S Toubi E Asher I Sthoeger Z Omalizumab for severe chronic spontaneous urticaria: Real-life experiences of 280 patients. J Allergy Clin Immunol Pract. 2017; 5: 1743–1745. 28988786 10.1016/j.jaip.2017.08.035

[22] Ghazanfar MN Sand C Thomsen SF Effectiveness and safety of omalizumab in chronic spontaneous or inducible urticaria: evaluation of 154 patients. Br J Dermatol. 2016; 175: 404–406. 26972689 10.1111/bjd.14540

[23] Zhao ZT Ji CM Yu WJ Meng L Hawro T Wei JF Maurer M Omalizumab for the treatment of chronic spontaneous urticaria: A meta-analysis of randomized clinical trials. J Allergy Clin Immunol. 2016; 137: 1742–1750.e4. 27040372 10.1016/j.jaci.2015.12.1342

[24] Papaianni V Guarneri F Vaccaro M Borgia F Guarneri C Cannavò SP From regulatory limitations to new opportunities: Real-life experience on the effectiveness of short courses of omalizumab in the treatment of chronic idiopatic urticaria. Dermatol Ther (Heidelb). 2020; 33: e13188 10.1111/dth.13188 31837248

[25] Salman A Comert E The real-life effectiveness and safety of omalizumab updosing in patients with chronic spontaneous urticaria. J Cutan Med Surg. 2019; 23: 496–500. 31030540 10.1177/1203475419847956

[26] Dressler C Werner RN Eisert L Zuberbier T Nast A Maurer M Chronic inducible urticaria: A systematic review of treatment options. J Allergy Clin Immunol. 2018; 141: 1726–1734. 29438771 10.1016/j.jaci.2018.01.031

[27] Kessel A Helou W Bamberger E Sabo E Nusem D Panassof J Toubi E Elevated serum total IgE – a potential marker for severe chronic urticaria. Int Arch Allergy Immunol. 2010; 153: 288–293. 20484928 10.1159/000314370

[28] Sánchez-Borges M Capriles-Hulet A Caballero-Fonseca F González-Aveledo L Justification for IgE as a therapeutic target in chronic spontaneous urticaria. Eur Ann Allergy Clin Immunol. 2017; 49: 148–153. 28752717 10.23822/eurannaci.1764-1489.02

[29] Gasser P Tarchevskaya SS Guntern P Brigger D Ruppli R Zbären N Kleinboelting S Heusser C Jardetzky TS Eggel A The mechanistic and functional profile of the therapeutic anti-IgE antibody ligelizumab differs from omalizumab. Nat Commun. 2020; 11: 165 31913280 10.1038/s41467-019-13815-w PMC6949303

[30] Simpson EL Bieber T Guttman-Yassky E Beck LA Blauvelt A Cork MJ Silverberg JI Deleuran M Kataoka Y Lacour JP Kingo K Worm M Poulin Y Wollenberg A Soo Y Graham NMH Pirozzi G Akinlade B Staudinger H Mastey V Two phase 3 trials of dupilumab versus placebo in atopic dermatitis. N Engl J Med. 2016; 375: 2335–2348. 27690741 10.1056/NEJMoa1610020

[31] Blauvelt A de Bruin-Weller M Gooderham M Cather JC Weisman J Pariser D Simpson EL Papp KA Hong HCH Rubel D Foley P Prens E Griffiths CEM Etoh T Pinto PH Pujol RM Szepietowski JC Ettler K Kemény L Zhu X Long-term management of moderate-to-severe atopic dermatitis with dupilumab and concomitant topical corticosteroids (LIBERTY AD CHRONOS): a 1-year, randomised, double-blinded, placebo-controlled, phase 3 trial. Lancet. 2017; 389: 2287–2303. 28478972 10.1016/S0140-6736(17)31191-1

[32] Simpson EL Paller AS Siegfried EC Boguniewicz M Sher L Gooderham MJ Beck LA Guttman-Yassky E Pariser D Blauvelt A Weisman J Lockshin B Hultsch T Zhang Q Kamal MA Davis JD Akinlade B Staudinger H Hamilton JD Graham NMH Efficacy and safety of dupilumab in adolescents with uncontrolled moderate to severe atopic dermatitis. JAMA Dermatol. 2020; 156: 44–56. 31693077 10.1001/jamadermatol.2019.3336 PMC6865265

[33] Paller AS Siegfried EC Thaçi D Wollenberg A Cork MJ Arkwright PD Gooderham M Beck LA Boguniewicz M Sher L Weisman J O’Malley JT Patel N Hardin M Graham NMH Ruddy M Sun X Davis JD Kamal MA Khokhar FA Efficacy and safety of dupilumab with concomitant topical corticosteroids in children 6 to 11 years old with severe atopic dermatitis: A randomized, double-blinded, placebo-controlled phase 3 trial. J Am Acad Dermatol. 2020; 83: 1282–1293. 32574587 10.1016/j.jaad.2020.06.054

[34] Werfel T Heratizadeh A Aberer W Ahrens F Augustin M Biedermann T Diepgen T Fölster-Holst R Kahle J Kapp A Nemat K Ott H Peters E Schlaeger M Schmid-Grendelmeier P Schmitt J Schwennesen T Staab D Traidl-Hoffmann C Werner R Aktualisierung „Systemtherapie bei Neurodermitis“ zur 2 Leitlinie Neurodermitis [atopisches Ekzem; atopische Dermatitis]. AWMF online: AWMF 4 Leitlinienregister Nr. 013/027, 2020: S2k., JDDG 2020 (in print). 2020; https://www.awmf.org/fileadmin/user_upload/Leitlinien/013_D_Dermatologische_Ges/013-027l_S2k_Neurodermitis_Aktualisierung-Systemtherapie_2020-06.pdf

[35] Heratizadeh A Haufe E Stölzl D Abraham S Heinrich L Kleinheinz A Wollenberg A Weisshaar E Augustin M Wiemers F Zink A von Kiedrowski R Hilgers M Worm M Pawlak M Sticherling M Fell I Handrick C Schäkel K Staubach-Renz P Baseline characteristics, disease severity and treatment history of patients with atopic dermatitis included in the German AD Registry TREATgermany. J Eur Acad Dermatol Venereol. 2020; 34: 1263–1272. 31721316 10.1111/jdv.16078

[36] Abraham S Haufe E Harder I Heratizadeh A Kleinheinz A Wollenberg A Weisshaar E Augustin M Wiemers F Zink A Biedermann T von Kiedrowski R Hilgers M Worm M Pawlak M Sticherling M Fell I Handrick C Schäkel K Staubach P Implementation of dupilumab in routine care of atopic eczema: results from the German national registry TREATgermany. Br J Dermatol. 2020; 183: 382–384. 32068242 10.1111/bjd.18958

[37] Wohlrab J Werfel T Wollenberg A Pathomechanism of dupilumab-associated inflammatory eye symptoms. J Eur Acad Dermatol Venereol. 2019; 33: e435–e436. 31220376 10.1111/jdv.15755

[38] Wohlrab J Wollenberg A Reimann H Pleyer U Werfel T [Interdisciplinary recommendations for action in dupilumab-related inflammatory eye diseases]. Hautarzt. 2019; 70: 64–67. 10.1007/s00105-018-4316-1 30478601

[39] Fleming P Drucker AM Risk of infection in patients with atopic dermatitis treated with dupilumab: A meta-analysis of randomized controlled trials. J Am Acad Dermatol. 2018; 78: 62–69.e1. 28987493 10.1016/j.jaad.2017.09.052

[40] Eichenfield LF Bieber T Beck LA Simpson EL Thaçi D de Bruin-Weller M Deleuran M Silverberg JI Ferrandiz C Fölster-Holst R Chen Z Graham NMH Pirozzi G Akinlade B Yancopoulos GD Ardeleanu M Infections in Dupilumab clinical trials in atopic dermatitis: A comprehensive pooled analysis. Am J Clin Dermatol. 2019; 20: 443–456. 31066001 10.1007/s40257-019-00445-7 PMC6533236

[41] Honstein T Werfel T The show must go on: an update on clinical experiences and clinical studies on novel pharmaceutical developments for the treatment of atopic dermatitis. Curr Opin Allergy Clin Immunol. 2020; 20: 386–394. 32452891 10.1097/ACI.0000000000000652

[42] Fokkens WJ Lund VJ Mullol J Bachert C Alobid I Baroody F Cohen N Cervin A Douglas R Gevaert P Georgalas C Goossens H Harvey R Hellings P Hopkins C Jones N Joos G Kalogjera L Kern B Kowalski M European position paper on rhinosinusitis and nasal polyps 2012. Rhinol Suppl. 2012; 23: 1–298. 22764607

[43] Hastan D Fokkens WJ Bachert C Newson RB Bislimovska J Bockelbrink A Bousquet PJ Brozek G Bruno A Dahlén SE Forsberg B Gunnbjörnsdóttir M Kasper L Krämer U Kowalski ML Lange B Lundbäck B Salagean E Todo-Bom A Tomassen P Chronic rhinosinusitis in Europe – an underestimated disease. A GA2LEN study. Allergy. 2011; 66: 1216–1223. 21605125 10.1111/j.1398-9995.2011.02646.x

[44] Rosenfeld RM Clinical practice guideline on adult sinusitis. Otolaryngol Head Neck Surg. 2007; 137: 365–377. 17765760 10.1016/j.otohns.2007.07.021

[45] Stuck BA Beule A Jobst D Klimek L Laudien M Lell M Vogl TJ Popert U [Guideline for “rhinosinusitis”-long version: S2k guideline of the German College of General Practitioners and Family Physicians and the German Society for Oto-Rhino-Laryngology, Head and Neck Surgery]. HNO. 2018; 66: 38–74. 28861645 10.1007/s00106-017-0401-5

[46] Calus L Van Zele T Derycke L Krysko O Dutre T Tomassen P Dullaers M Bachert C Gevaert P Local inflammation in chronic upper airway disease. Curr Pharm Des. 2012; 18: 2336–2346. 22390697 10.2174/138161212800166022

[47] Grundmann SA Hemfort PB Luger TA Brehler R Anti-IgE (omalizumab): a new therapeutic approach for chronic rhinosinusitis. J Allergy Clin Immunol. 2008; 121: 257–258. 18206513 10.1016/j.jaci.2007.09.036

[48] Holgate ST Djukanović R Casale T Bousquet J Anti-immunoglobulin E treatment with omalizumab in allergic diseases: an update on anti-inflammatory activity and clinical efficacy. Clin Exp Allergy. 2005; 35: 408–416. 15836747 10.1111/j.1365-2222.2005.02191.x

[49] Pinto JM Mehta N DiTineo M Wang J Baroody FM Naclerio RM A randomized, double-blind, placebo-controlled trial of anti-IgE for chronic rhinosinusitis. Rhinology. 2010; 48: 318–324. 21038023 10.4193/Rhino09.144

[50] Vennera MDC Sabadell C Picado C Duration of the efficacy of omalizumab after treatment discontinuation in “real life” severe asthma. Thorax. 2018; 73: 782–784. 29079610 10.1136/thoraxjnl-2017-210017

[51] Gevaert P Calus L Van Zele T Blomme K De Ruyck N Bauters W Hellings P Brusselle G De Bacquer D van Cauwenberge P Bachert C Omalizumab is effective in allergic and non-allergic patients with nasal polyps and asthma. J Allergy Clin Immunol. 2013; 131: 110–6 e1. 23021878 10.1016/j.jaci.2012.07.047

[52] Subcutaneuous omalizumab for treatment of chronic rhinosinusitis with nassal polyposis (Xolair CRS) [Available from: https://clinicaltrials.gov/ct/show/NCT01066104.

[53] Gevaert P Omachi TA Corren J Mullol J Han J Lee SE Kaufman D Ligueros-Saylan M Howard M Zhu R Owen R Wong K Islam L Bachert C Efficacy and safety of omalizumab in nasal polyposis: 2 randomized phase 3 trials. J Allergy Clin Immunol. 2020; 146: 595–605. 32524991 10.1016/j.jaci.2020.05.032

[54] Flood-Page P Swenson C Faiferman I Matthews J Williams M Brannick L Robinson D Wenzel S Busse W Hansel TT Barnes NC A study to evaluate safety and efficacy of mepolizumab in patients with moderate persistent asthma. Am J Respir Crit Care Med. 2007; 176: 1062–1071. 17872493 10.1164/rccm.200701-085OC

[55] Flood-Page PT Menzies-Gow AN Kay AB Robinson DS Eosinophil’s role remains uncertain as anti-interleukin-5 only partially depletes numbers in asthmatic airway. Am J Respir Crit Care Med. 2003; 167: 199–204. 12406833 10.1164/rccm.200208-789OC

[56] Bel EH Wenzel SE Thompson PJ Prazma CM Keene ON Yancey SW Ortega HG Pavord ID Oral glucocorticoid-sparing effect of mepolizumab in eosinophilic asthma. N Engl J Med. 2014; 371: 1189–1197. 25199060 10.1056/NEJMoa1403291

[57] Gevaert P Van Bruaene N Cattaert T Van Steen K Van Zele T Acke F De Ruyck N Blomme K Sousa AR Marshall RP Bachert C Mepolizumab, a humanized anti-IL-5 mAb, as a treatment option for severe nasal polyposis. J Allergy Clin Immunol. 2011; 128: 989–95 e1-8. 21958585 10.1016/j.jaci.2011.07.056

[58] Mepolizumab in nasal polyposis. [Available from: https://clinicaltrials.gov/ct2/show/NCT01362244].

[59] Effect of mepolizumab in severe bilateral nasal polyps. [Available from: https://clinicaltrials.gov/ct2/show/NCT03085797].

[60] Castro M Zangrilli J Wechsler ME Bateman ED Brusselle GG Bardin P Murphy K Maspero JF O’Brien C Korn S Reslizumab for inadequately controlled asthma with elevated blood eosinophil counts: results from two multicentre, parallel, double-blind, randomised, placebo-controlled, phase 3 trials. Lancet Respir Med. 2015; 3: 355–366. 25736990 10.1016/S2213-2600(15)00042-9

[61] Castro M Mathur S Hargreave F Boulet LP Xie F Young J Wilkins HJ Henkel T Nair P Reslizumab for poorly controlled, eosinophilic asthma: a randomized, placebo-controlled study. Am J Respir Crit Care Med. 2011; 184: 1125–1132. 21852542 10.1164/rccm.201103-0396OC

[62] Gevaert P Lang-Loidolt D Lackner A Stammberger H Staudinger H Van Zele T Holtappels G Tavernier J van Cauwenberge P Bachert C Nasal IL-5 levels determine the response to anti-IL-5 treatment in patients with nasal polyps. J Allergy Clin Immunol. 2006; 118: 1133–1141. 17088140 10.1016/j.jaci.2006.05.031

[63] Laviolette M Gossage DL Gauvreau G Leigh R Olivenstein R Katial R Busse WW Wenzel S Wu Y Datta V Kolbeck R Molfino NA Effects of benralizumab on airway eosinophils in asthmatic patients with sputum eosinophilia. J Allergy Clin Immunol. 2013; 132: 1086–96 e5. 23866823 10.1016/j.jaci.2013.05.020 PMC4172321

[64] Efficacy and safety study of benralizumab for patients with severe nasal polyposis (OSTRO). [Available from: https://clinicaltrials.gov/ct2/show/NCT03401229].

[65] Wenzel S Castro M Corren J Maspero J Wang L Zhang B Pirozzi G Sutherland ER Evans RR Joish VN Eckert L Graham NM Stahl N Yancopoulos GD Louis-Tisserand M Teper A Dupilumab efficacy and safety in adults with uncontrolled persistent asthma despite use of medium-to-high-dose inhaled corticosteroids plus a long-acting b2 agonist: a randomised double-blind placebo-controlled pivotal phase 2b dose-ranging trial. Lancet. 2016; 388: 31–44. 27130691 10.1016/S0140-6736(16)30307-5

[66] Wenzel SE Wang L Pirozzi G Dupilumab in persistent asthma. N Engl J Med. 2013; 369: 1276 24066755 10.1056/NEJMc1309809

[67] Wechsler ME Inhibiting interleukin-4 and interleukin-13 in difficult-to-control asthma. N Engl J Med. 2013; 368: 2511–2513. 23688322 10.1056/NEJMe1305426

[68] Bachert C Mannent L Naclerio RM Mullol J Ferguson BJ Gevaert P Hellings P Jiao L Wang L Evans RR Pirozzi G Graham NM Swanson B Hamilton JD Radin A Gandhi NA Stahl N Yancopoulos GD Sutherland ER Effect of subcutaneous dupilumab on nasal polyp burden in patients with chronic sinusitis and nasal Polyposis: A randomized clinical trial JAMA. 2016; 315: 469–479. 26836729 10.1001/jama.2015.19330

[69] Bachert C Han JK Desrosiers M Hellings PW Amin N Lee SE Mullol J Greos LS Bosso JV Laidlaw TM Cervin AU Maspero JF Hopkins C Olze H Canonica GW Paggiaro P Cho SH Fokkens WJ Fujieda S Zhang M Efficacy and safety of dupilumab in patients with severe chronic rhinosinusitis with nasal polyps (LIBERTY NP SINUS-24 and LIBERTY NP SINUS-52): results from two multicentre, randomised, double-blind, placebo-controlled, parallel-group phase 3 trials. Lancet. 2019; 394: 1638–1650. 31543428 10.1016/S0140-6736(19)31881-1

[70] Magerl M Brasch J Förster U Hauswald B Mohr EB Prässler J Treudler R Vetter R Wahn V Zampeli V Ziemer M Maurer M [Diagnostics and exclusion of hereditary angioedema: a standarized approach for the practice]. Hautarzt. 2012; 63: 567–572. 22751857 10.1007/s00105-012-2388-x

[71] Maurer M Magerl M Ansotegui I Aygören-Pürsün E Betschel S Bork K Bowen T Balle Boysen H Farkas H Grumach AS Hide M Katelaris C Lockey R Longhurst H Lumry WR Martinez-Saguer I Moldovan D Nast A Pawankar R Potter P The international WAO/EAACI guideline for the management of hereditary angioedema-The 2017 revision and update. Allergy. 2018; 73: 1575–1596. 29318628 10.1111/all.13384

[72] Syed YY Lanadelumab: first global approval. Drugs. 2018; 78: 1633–1637. 30267321 10.1007/s40265-018-0987-2

[73] Riedl MA Bernstein JA Craig T Banerji A Magerl M Cicardi M Longhurst HJ Shennak MM Yang WH Schranz J Baptista J Busse PJ An open-label study to evaluate the long-term safety and efficacy of lanadelumab for prevention of attacks in hereditary angioedema: design of the HELP study extension. Clin Transl Allergy. 2017; 7: 36 29043014 10.1186/s13601-017-0172-9 PMC5629784

[74] Banerji A Busse P Shennak M Lumry W Davis-Lorton M Wedner HJ Jacobs J Baker J Bernstein JA Lockey R Li HH Craig T Cicardi M Riedl M Al-Ghazawi A Soo C Iarrobino R Sexton DJ TenHoor C Kenniston JA Inhibiting plasma kallikrein for hereditary angioedema prophylaxis. N Engl J Med. 2017; 376: 717–728. 28225674 10.1056/NEJMoa1605767

[75] Riedl MA Maurer M Bernstein JA Banerji A Longhurst HJ Li HH Lu P Hao J Juethner S Lumry WR Hébert J Ritchie B Sussman G Yang WH Escuriola Ettingshausen C Magerl M Martinez-Saguer I Maurer M Staubach P Zimmer S Lanadelumab demonstrates rapid and sustained prevention of hereditary angioedema attacks. Allergy. 2020; 75: 2879–2887. 32452549 10.1111/all.14416 PMC7689768

[76] Craig T Magerl M Levy DS Rehef A Lumry WL Martinzes-Saguer I Jacobs JS Yang WH Ritchie B Aygören-Pursün E Keith PK Busse P Feuersenger H Jacobs I Pragst I Results of a randomized, double-blind, placebo-controlled, phase 2 study, investigating the safety and efficacy of anti-factor XIIa monoclonal antibody garadacimab (CSL312) for prophylaxis of HAE. 2020.

[77] Sampson HA Leung DY Burks AW Lack G Bahna SL Jones SM Wong DA A phase II, randomized, doubleblind, parallelgroup, placebocontrolled oral food challenge trial of Xolair (omalizumab) in peanut allergy. J Allergy Clin Immunol. 2011; 127: 1309–10.e1. 21397314 10.1016/j.jaci.2011.01.051

[78] Schneider LC Rachid R LeBovidge J Blood E Mittal M Umetsu DT A pilot study of omalizumab to facilitate rapid oral desensitization in high-risk peanut-allergic patients. J Allergy Clin Immunol. 2013; 132: 1368–1374. 24176117 10.1016/j.jaci.2013.09.046 PMC4405160

[79] Schocker F Recke A Kull S Worm M Jappe U Persistent cow′s milk anaphylaxis from early childhood monitored by IgE and BAT to cow′s and human milk under therapy. Pediatr Allergy Immunol. 2018; 29: 210–214. 29197130 10.1111/pai.12843

[80] Nadeau KC Schneider LC Hoyte L Borras I Umetsu DT Rapid oral desensitization in combination with omalizumab therapy in patients with cow’s milk allergy. J Allergy Clin Immunol. 2011; 127: 1622–1624. 21546071 10.1016/j.jaci.2011.04.009 PMC3396422

[81] Lafuente I Mazon A Nieto M Uixera S Pina R Nieto A Possible recurrence of symptoms after discontinuation of omalizumab in anti-IgE-assisted desensitization to egg. Pediatr Allergy Immunol. 2014; 25: 717–719. 24902874 10.1111/pai.12259

[82] MacGinnitie AJ Rachid R Gragg H Little SV Lakin P Cianferoni A Heimall J Makhija M Robison R Chinthrajah RS Lee J Lebovidge J Dominguez T Rooney C Lewis MO Koss J Burke-Roberts E Chin K Logvinenko T Pongracic JA Omalizumab facilitates rapid oral desensitization for peanut allergy. J Allergy Clin Immunol. 2017; 139: 873–881.e8. 27609658 10.1016/j.jaci.2016.08.010 PMC5369605

[83] Andorf S Manohar M Dominguez T Block W Tupa D Kshirsagar RA Sampath V Chinthrajah RS Nadeau KC Observational long-term follow-up study of rapid food oral immunotherapy with omalizumab. Allergy Asthma Clin Immunol. 2017; 13: 53 10.1186/s13223-017-0223-8 PMC5738812 29296107

[84] Crisafulli G Caminiti L Chiera F Arasi S Salzano G Panasiti I Barbalace A Pajno GB Omalizumab in children with severe allergic disease: a case series. Ital J Pediatr. 2019; 45: 13 30642367 10.1186/s13052-019-0602-5 PMC6332555

[85] Worm M Reese I Ballmer-Weber B Beyer K Bischoff SC Classen M Fischer PJ Fuchs T Huttegger I Jappe U Klimek L Koletzko B Lange L Lepp U Mahler V Niggemann B Rabe U Raithel M Saloga J Schäfer C Guidelines on the management of IgE-mediated food allergies: S2k-Guidelines of the German Society for Allergology and Clinical Immunology (DGAKI) in collaboration with the German Medical Association of Allergologists (AeDA), the German Professional Association of Pediatricians (BVKJ), the German Allergy and Asthma Association (DAAB), German Dermatological Society (DDG), the German Society for Nutrition (DGE), the German Society for Gastroenterology, Digestive and Metabolic Diseases (DGVS), the German Society for Oto-Rhino-Laryngology, Head and Neck Surgery, the German Society for Pediatric and Adolescent Medicine (DGKJ), the German Society for Pediatric Allergology and Environmental Medicine (GPA), the German Society for Pneumology (DGP), the German Society for Pediatric Gastroenterology and Nutrition (GPGE), German Contact Allergy Group (DKG), the Austrian Society for Allergology and Immunology (Æ-GAI), German Professional Association of Nutritional Sciences (VDOE) and the Association of the Scientific Medical Societies Germany (AWMF). Allergo J Int. 2015; 24: 256–293. 27069841 10.1007/s40629-015-0074-0 PMC4792347

[86] Pichler WJ Adverse side-effects to biological agents. Allergy. 2006; 61: 912–920. 16867042 10.1111/j.1398-9995.2006.01058.x

[87] Joshi SR Khan DA Anaphylaxis induced by biologics. Curr Treat Options Allergy. 2019; 6: 125–141.

[88] Gülsen A Wedi B Jappe U Hypersensitivity reactions to biologics (part I): allergy as an important differential diagnosis in complex immune-derived adverse events. Allergo J Int. 2020; 29: 1–29. 10.1007/s15007-020-2550-1 PMC7289641 32546899

[89] Gülsen A Wedi B Jappe U Hypersensitivity reactions to biologics (part II): Classifications and current diagnostic and treatment approaches. Allergo J Int. 2020; 29: 139–154. 10.1007/s40629-020-00126-6 PMC7223134 32421085

[90] Chung CH Mirakhur B Chan E Le QT Berlin J Morse M Murphy BA Satinover SM Hosen J Mauro D Slebos RJ Zhou Q Gold D Hatley T Hicklin DJ Platts-Mills TA Cetuximab-induced anaphylaxis and IgE specific for galactose-alpha-1,3-galactose. N Engl J Med. 2008; 358: 1109–1117. 18337601 10.1056/NEJMoa074943 PMC2361129

[91] Chitnavis M Stein DJ Commins S Schuyler AJ Behm B First-dose anaphylaxis to infliximab: a case of mammalian meat allergy. J Allergy Clin Immunol Pract. 2017; 5: 1425–1426. 28634101 10.1016/j.jaip.2017.04.044

[92] Vultaggio A Matucci A Nencini F Pratesi S Parronchi P Rossi O Romagnani S Maggi E Anti-infliximab IgE and non-IgE antibodies and induction of infusion-related severe anaphylactic reactions. Allergy. 2010; 65: 657–661. 19951375 10.1111/j.1398-9995.2009.02280.x

[93] Matucci A Pratesi S Petroni G Nencini F Virgili G Milla M Maggi E Vultaggio A Allergological in vitro and in vivo evaluation of patients with hypersensitivity reactions to infliximab. Clin Exp Allergy. 2013; 43: 659–664. 23711128 10.1111/cea.12098

[94] Svenson M Geborek P Saxne T Bendtzen K Monitoring patients treated with anti-TNF-alpha biopharmaceuticals: assessing serum infliximab and anti-infliximab antibodies. Rheumatology (Oxford). 2007; 46: 1828–1834. 18032541 10.1093/rheumatology/kem261

[95] Hwang SH Yoo H-S Yoon MK Park H-S Detection of IgE binding component to infliximab in a patient with infliximab-induced anaphylaxis. Ann Allergy Asthma Immunol. 2014; 112: 393–394. 24583135 10.1016/j.anai.2014.02.001

[96] Weiss J Grilley Olson J Deal AM Chera B Weissler M Murphy BA Hayes DN Gilbert J Using the galactose-α-1,3-galactose enzyme-linked immunosorbent assay to predict anaphylaxis in response to cetuximab. Cancer. 2016; 122: 1697–1701. 26989991 10.1002/cncr.29978

[97] Corominas M Gastaminza G Lobera T Hypersensitivity reactions to biological drugs. J Investig Allergol Clin Immunol. 2014; 24: 212–225, quiz 1p, 225. 25219103

[98] Treudler R Delaroque N Puder M Simon JC Szardenings M Dupilumab-induced serum sickness-like reaction: an unusual adverse effect in a patient with atopic eczema. J Eur Acad Dermatol Venereol. 2020; epub ahead of print. 10.1111/jdv.16782 32594596

[99] Homann A Röckendorf N Kromminga A Frey A Jappe U Immunogenic infliximab epitopes located in TNF-alpha binding regions with no cross-reactivity to adalimumab. J Transl Med. 2015; 13: 339 26511203 10.1186/s12967-015-0706-7 PMC4625721

[100] Homann A Röckendorf N Kromminga A Frey A Platts-Mills TA Jappe U Glycan and peptide IgE epitopes of the TNF-alpha blockers infliximab and adalimumab – precision diagnostics by cross-reactivity immune profiling of patient sera. Theranostics. 2017; 7: 4699–4709. 29187897 10.7150/thno.20654 PMC5706093

[101] Abreu C Sarmento A Magro F Screening, prophylaxis and counselling before the start of biological therapies: A practical approach focused on IBD patients. Dig Liver Dis. 2017; 49: 1289–1297. 28986117 10.1016/j.dld.2017.09.002

[102] Dobler CC Martin A Marks GB Benefit of treatment of latent tuberculosis infection in individual patients. Eur Respir J. 2016; 47: 1594–1595. 27132273 10.1183/13993003.00175-2016

[103] Blauvelt A Simpson EL Tyring SK Purcell LA Shumel B Petro CD Akinlade B Gadkari A Eckert L Graham NMH Pirozzi G Evans R Dupilumab does not affect correlates of vaccine-induced immunity: A randomized, placebo-controlled trial in adults with moderate-to-severe atopic dermatitis. J Am Acad Dermatol. 2019; 80: 158–167.e1. 30092324 10.1016/j.jaad.2018.07.048

[104] Zeitlin PL Leong M Cole J Mallory RM Shih VH Olsson RF Goldman M Benralizumab does not impair antibody response to seasonal influenza vaccination in adolescent and young adult patients with moderate to severe asthma: results from the Phase IIIb ALIZE trial. J Asthma Allergy. 2018; 11: 181–192. 30510434 10.2147/JAA.S172338 PMC6248228

[105] Østensen M The use of biologics in pregnant patients with rheumatic disease. Expert Rev Clin Pharmacol. 2017; 10: 661–669. 28326845 10.1080/17512433.2017.1305268

[106] Brouwer J Hazes JM Laven JS Dolhain RJ Fertility in women with rheumatoid arthritis: influence of disease activity and medication. Ann Rheum Dis. 2015; 74: 1836–1841. 24833784 10.1136/annrheumdis-2014-205383

[107] Ostensen M Safety issues of biologics in pregnant patients with rheumatic diseases. Ann N Y Acad Sci. 2014; 1317: 32–38. 24840548 10.1111/nyas.12456

[108] Götestam Skorpen C Hoeltzenbein M Tincani A Fischer-Betz R Elefant E Chambers C da Silva J Nelson-Piercy C Cetin I Costedoat-Chalumeau N Dolhain R Förger F Khamashta M Ruiz-Irastorza G Zink A Vencovsky J Cutolo M Caeyers N Zumbühl C Østensen M The EULAR points to consider for use of antirheumatic drugs before pregnancy, and during pregnancy and lactation. Ann Rheum Dis. 2016; 75: 795–810. 26888948 10.1136/annrheumdis-2015-208840

[109] Flint J Panchal S Hurrell A van de Venne M Gayed M Schreiber K Arthanari S Cunningham J Flanders L Moore L Crossley A Purushotham N Desai A Piper M Nisar M Khamashta M Williams D Gordon C Giles I BSR and BHPR guideline on prescribing drugs in pregnancy and breastfeeding-Part I: standard and biologic disease modifying anti-rheumatic drugs and corticosteroids. Rheumatology (Oxford). 2016; 55: 1693–1697. 26750124 10.1093/rheumatology/kev404

[110] Gerosa M Argolini LM Artusi C Chighizola CB The use of biologics and small molecules in pregnant patients with rheumatic diseases. Expert Rev Clin Pharmacol. 2018; 11: 987–998. 30227748 10.1080/17512433.2018.1525293

[111] Namazy JA Blais L Andrews EB Scheuerle AE Cabana MD Thorp JM Umetsu DT Veith JH Sun D Kaufman DG Covington DL Mukhopadhyay S Fogel RB Lopez-Leon S Spain CV Pregnancy outcomes in the omalizumab pregnancy registry and a disease-matched comparator cohort. J Allergy Clin Immunol. 2020; 145: 528–536.e1. 31145939 10.1016/j.jaci.2019.05.019

[112] Klimek L Pfaar O Worm M Eiwegger T Hagemann J Ollert M Untersmayr E Hoffmann-Sommergruber K Vultaggio A Agache I Bavbek S Bossios A Casper I Chan S Chatzipetrou A Vogelberg C Firinu D Kauppi P Kolios A Kothari A Use of biologicals in allergic and type-2 inflammatory diseases during the current COVID-19 pandemic. Position paper of Ärzteverband Deutscher Allergologen (AeDA), Deutsche Gesellschaft für Allergologie und Klinische Immunologie (DGAKI), Gesellschaft für Pädiatrische Allergologie und Umweltmedizin (GPA), Österreichische Gesellschaft für Allergologie und Immunologie (ÖGAI), Luxemburgische Gesellschaft für Allergologie und Immunologie (LGAI), Österreichische Gesellschaft für Pneumologie (ÖGP) in co-operation with the German, Austrian, and Swiss ARIA groups, and the European Academy of Allergy and Clinical Immunology (EAACI) 2020; 4: 53–68. 10.5414/ALX02166E PMC7480069 32915172

[113] Cork MJ Thaçi D Eichenfield LF Arkwright PD Hultsch T Davis JD Zhang Y Zhu X Chen Z Li M Ardeleanu M Teper A Akinlade B Gadkari A Eckert L Kamal MA Ruddy M Graham NMH Pirozzi G Stahl N Dupilumab in adolescents with uncontrolled moderate-to-severe atopic dermatitis: results from a phase IIa open-label trial and subsequent phase III open-label extension. Br J Dermatol. 2020; 182: 85–96. 31595499 10.1111/bjd.18476 PMC6972638

[114] de Bruin-Weller M Thaçi D Smith CH Reich K Cork MJ Radin A Zhang Q Akinlade B Gadkari A Eckert L Hultsch T Chen Z Pirozzi G Graham NMH Shumel B Dupilumab with concomitant topical corticosteroid treatment in adults with atopic dermatitis with an inadequate response or intolerance to ciclosporin A or when this treatment is medically inadvisable: a placebo-controlled, randomized phase III clinical trial (LIBERTY AD CAFÉ). Br J Dermatol. 2018; 178: 1083–1101. 29193016 10.1111/bjd.16156

[115] Wollenberg A Blauvelt A Guttman‐Yassky E Worm M Lynde C Lacour JP Spelman L Katoh N Saeki H Poulin Y Lesiak A Kircik L Cho SH Herranz P Cork MJ Peris K Steffensen LA Bang B Kuznetsova A Jensen TN Østerdal ML Simpson EL Tralokinumab for moderate‐to‐severe atopic dermatitis: results from two 52‐week, randomized, double‐blind, multicentre, placebo‐controlled phase III trials (ECZTRA 1 and ECZTRA 2). Br J Dermatol. 2020; epub ahead of print. 10.1111/bjd.19574 PMC7986411 33000465

[116] Simpson EL Lacour JP Spelman L Galimberti R Eichenfield LF Bissonnette R King BA Thyssen JP Silverberg JI Bieber T Kabashima K Tsunemi Y Costanzo A Guttman-Yassky E Beck LA Janes JM DeLozier AM Gamalo M Brinker DR Cardillo T Baricitinib in patients with moderate-to-severe atopic dermatitis and inadequate response to topical corticosteroids: results from two randomized monotherapy phase III trials. Br J Dermatol. 2020; 183: 242–255. 31995838 10.1111/bjd.18898

[117] Simpson EL Flohr C Eichenfield LF Bieber T Sofen H Taïeb A Owen R Putnam W Castro M DeBusk K Lin CY Voulgari A Yen K Omachi TA Efficacy and safety of lebrikizumab (an anti-IL-13 monoclonal antibody) in adults with moderate-to-severe atopic dermatitis inadequately controlled by topical corticosteroids: A randomized, placebo-controlled phase II trial (TREBLE). J Am Acad Dermatol. 2018; 78: 863–871.e11. 29353026 10.1016/j.jaad.2018.01.017

[118] Guttman-Yassky E Brunner PM Neumann AU Khattri S Pavel AB Malik K Singer GK Baum D Gilleaudeau P Sullivan-Whalen M Rose S Jim On S Li X Fuentes-Duculan J Estrada Y Garcet S Traidl-Hoffmann C Krueger JG Lebwohl MG Efficacy and safety of fezakinumab (an IL-22 monoclonal antibody) in adults with moderate-to-severe atopic dermatitis inadequately controlled by conventional treatments: A randomized, double-blind, phase 2a trial. J Am Acad Dermatol. 2018; 78: 872–81. e6. 29353025 10.1016/j.jaad.2018.01.016 PMC8711034

[119] Gittler JK Shemer A Suárez-Fariñas M Fuentes-Duculan J Gulewicz KJ Wang CQF Mitsui H Cardinale I de Guzman Strong C Krueger JG Guttman-Yassky E Progressive activation of T(H)2/T(H)22 cytokines and selective epidermal proteins characterizes acute and chronic atopic dermatitis. J Allergy Clin Immunol. 2012; 130: 1344–1354. 22951056 10.1016/j.jaci.2012.07.012 PMC3991245

[120] Kabashima K Furue M Hanifin JM Pulka G Wollenberg A Galus R Etoh T Mihara R Nakano M Ruzicka T Nemolizumab in patients with moderate-to-severe atopic dermatitis: Randomized, phase II, long-term extension study. J Allergy Clin Immunol. 2018; 142: 1121–1130.e7. 29753033 10.1016/j.jaci.2018.03.018

[121] Chen YL Gutowska-Owsiak D Hardman CS Westmoreland M MacKenzie T Cifuentes L Waithe D Lloyd-Lavery A Marquette A Londei M Ogg G Proof-of-concept clinical trial of etokimab shows a key role for IL-33 in atopic dermatitis pathogenesis. Sci Transl Med. 2019; 11: eaax2945. 31645451 10.1126/scitranslmed.aax2945

[122] Klimek L Förster-Ruhrmann U Becker S Chaker A Strieth S Hoffmann TK Dazert S Deitmer T Olze H Glien A Plontke S Wrede H Schlenter W Welkoborsky HJ Wollenberg B Beule AG Rudack C Wagenmann M Stöver T Huppertz T Hagemann J Bachert C Positionspapier: Anwendung von Biologika bei chronischer Rhinosinusitis mit Polyposis nasi (CRSwNP) im deutschen Gesundheitssystem. Empfehlungen des Ärzteverbandes Deutscher Allergologen (AeDA) und der AGs Klinische Immunologie, Allergologie und Umweltmedizin und Rhinologie und Rhinochirurgie der Deutschen Gesellschaft für HNO-Heilkunde, Kopf- und Halschirurgie (DGHNOKHC). Laryngorhinootologie. 2020; 99: 511–527. 32575138 10.1055/a-1197-0136

[123] Agarwal A Spath D Sherris DA Kita H Ponikau JU Therapeutic antibodies for nasal polyposis treatment: Where are we headed? Clin Rev Allergy Immunol. 2020; 59: 141–149. 31073812 10.1007/s12016-019-08734-z

[124] Wenzel S Severe asthma: from characteristics to phenotypes to endotypes. Clin Exp Allergy. 2012; 42: 650–658. 22251060 10.1111/j.1365-2222.2011.03929.x

[125] Godar M Blanchetot C de Haard H Lambrecht BN Brusselle G Personalized medicine with biologics for severe type 2 asthma: current status and future prospects. MAbs. 2018; 10: 34–45. 29035619 10.1080/19420862.2017.1392425 PMC5800381

[126] Farne HA Wilson A Powell C Bax L Milan SJ Anti-IL5 therapies for asthma. Cochrane Database Syst Rev. 2017; 9: CD010834. 28933516 10.1002/14651858.CD010834.pub3 PMC6483800

[127] Hammad H Lambrecht BN Barrier epithelial cells and the control of type 2 immunity. Immunity. 2015; 43: 29–40. 26200011 10.1016/j.immuni.2015.07.007

[128] Corren J Parnes JR Wang L Mo M Roseti SL Griffiths JM van der Merwe R Tezepelumab in adults with uncontrolled asthma. N Engl J Med. 2017; 377: 936–946. 28877011 10.1056/NEJMoa1704064

[129] Gauvreau GM O’Byrne PM Boulet L-P Wang Y Cockcroft D Bigler J FitzGerald JM Boedigheimer M Davis BE Dias C Gorski KS Smith L Bautista E Comeau MR Leigh R Parnes JR Effects of an anti-TSLP antibody on allergen-induced asthmatic responses. N Engl J Med. 2014; 370: 2102–2110. 24846652 10.1056/NEJMoa1402895

[130] Robinson D Humbert M Buhl R Cruz AA Inoue H Korom S Hanania NA Nair P Revisiting Type 2-high and Type 2-low airway inflammation in asthma: current knowledge and therapeutic implications. Clin Exp Allergy. 2017; 47: 161–175. 28036144 10.1111/cea.12880

[131] Busse WW Holgate S Kerwin E Chon Y Feng J Lin J Lin SL Randomized, double-blind, placebo-controlled study of brodalumab, a human anti-IL-17 receptor monoclonal antibody, in moderate to severe asthma. Am J Respir Crit Care Med. 2013; 188: 1294–1302. 24200404 10.1164/rccm.201212-2318OC

[132] Holgate ST Noonan M Chanez P Busse W Dupont L Pavord I Hakulinen A Paolozzi L Wajdula J Zang C Nelson H Raible D Efficacy and safety of etanercept in moderate-to-severe asthma: a randomised, controlled trial. Eur Respir J. 2011; 37: 1352–1359. 21109557 10.1183/09031936.00063510

[133] Wenzel SE Barnes PJ Bleecker ER Bousquet J Busse W Dahlén SE Holgate ST Meyers DA Rabe KF Antczak A Baker J Horvath I Mark Z Bernstein D Kerwin E Schlenker-Herceg R Lo KH Watt R Barnathan ES Chanez P A randomized, double-blind, placebo-controlled study of tumor necrosis factor-alpha blockade in severe persistent asthma. Am J Respir Crit Care Med. 2009; 179: 549–558. 19136369 10.1164/rccm.200809-1512OC

[134] Nair P Gaga M Zervas E Alagha K Hargreave FE O’Byrne PM Stryszak P Gann L Sadeh J Chanez P Safety and efficacy of a CXCR2 antagonist in patients with severe asthma and sputum neutrophils: a randomized, placebo-controlled clinical trial. Clin Exp Allergy. 2012; 42: 1097–1103. 22702508 10.1111/j.1365-2222.2012.04014.x

[135] O’Byrne PM Metev H Puu M Richter K Keen C Uddin M Larsson B Cullberg M Nair P Efficacy and safety of a CXCR2 antagonist, AZD5069, in patients with uncontrolled persistent asthma: a randomised, double-blind, placebo-controlled trial. Lancet Respir Med. 2016; 4: 797–806. 27574788 10.1016/S2213-2600(16)30227-2

[136] Cox L Platts-Mills TA Finegold I Schwartz LB Simons FE Wallace DV American Academy of Allergy, Asthma & Immunology/American College of Allergy, Asthma and Immunology Joint Task Force Report on omalizumab-associated anaphylaxis. J Allergy Clin Immunol. 2007; 120: 1373–1377. 17996286 10.1016/j.jaci.2007.09.032

[137] Di Bona D Fiorino I Taurino M Frisenda F Minenna E Pasculli C Kourtis G Rucco AS Nico A Albanesi M Giliberti L D’Elia L Caiaffa MF Macchia L Long-term “real-life” safety of omalizumab in patients with severe uncontrolled asthma: A nine-year study. Respir Med. 2017; 130: 55–60. 29206634 10.1016/j.rmed.2017.07.013

[138] US Food and Drug Administration. FDA labels for omalizumab (XOLAIR^®^). 2019. https://www.accessdata.fda.gov/drugsatfda_docs/label/2019/103976s5234lbl.pdf (Accessed June 9, 2020)

[139] European Medicines Agency. Assessment Report for omalizumab (XOLAIR^®^). 2019. https://www.ema.europa.eu/en/documents/product-information/xolair-epar-product-information_en.pdf.

[140] Gauvreau GM Arm JP Boulet LP Leigh R Cockcroft DW Davis BE Mayers I FitzGerald JM Dahlen B Killian KJ Laviolette M Carlsten C Lazarinis N Watson RM Milot J Swystun V Bowen M Hui L Lantz AS Meiser K Efficacy and safety of multiple doses of QGE031 (ligelizumab) versus omalizumab and placebo in inhibiting allergen-induced early asthmatic responses. J Allergy Clin Immunol. 2016; 138: 1051–1059. 27185571 10.1016/j.jaci.2016.02.027

[141] Maurer M Giménez-Arnau AM Sussman G Metz M Baker DR Bauer A Bernstein JA Brehler R Chu CY Chung WH Danilycheva I Grattan C Hébert J Katelaris C Makris M Meshkova R Savic S Sinclair R Sitz K Staubach P Ligelizumab for chronic spontaneous urticaria. N Engl J Med. 2019; 381: 1321–1332. 31577874 10.1056/NEJMoa1900408

[142] Pavord ID Korn S Howarth P Bleecker ER Buhl R Keene ON Ortega H Chanez P Mepolizumab for severe eosinophilic asthma (DREAM): a multicentre, double-blind, placebo-controlled trial. Lancet. 2012; 380: 651–659. 22901886 10.1016/S0140-6736(12)60988-X

[143] Lugogo N Domingo C Chanez P Leigh R Gilson MJ Price RG Yancey SW Ortega HG Long-term efficacy and safety of mepolizumab in patients with severe eosinophilic asthma: a multi-center, open-label, phase IIIb study. Clin Ther. 2016; 38: 2058–2070.e1. 27553751 10.1016/j.clinthera.2016.07.010

[144] Khatri S Moore W Gibson PG Leigh R Bourdin A Maspero J Barros M Buhl R Howarth P Albers FC Bradford ES Gilson M Price RG Yancey SW Ortega H Assessment of the long-term safety of mepolizumab and durability of clinical response in patients with severe eosinophilic asthma. J Allergy Clin Immunol. 2019; 143: 1742–1751.e7. 30359681 10.1016/j.jaci.2018.09.033

[145] US Food and Drug Administration. FDA labels for mepolizumab (Nucala^®^). 2019. https://www.accessdata.fda.gov/drugsatfda_docs/label/2019/125526s013lbl.pdf (Accessed June 10, 2020).

[146] European Medicines Agency. Assessment Report for mepolizumab (Nucala^®^). 2019. https://www.ema.europa.eu/en/documents/product-information/nucala-epar-product-information_en.pdf (Accessed June 10, 2020).

[147] Chapman KR Albers FC Chipps B Muñoz X Devouassoux G Bergna M Galkin D Azmi J Mouneimne D Price RG Liu MC The clinical benefit of mepolizumab replacing omalizumab in uncontrolled severe eosinophilic asthma. Allergy. 2019; 74: 1716–1726. 31049972 10.1111/all.13850 PMC6790683

[148] Murphy K Jacobs J Bjermer L Fahrenholz JM Shalit Y Garin M Zangrilli J Castro M Long-term safety and efficacy of reslizumab in patients with eosinophilic asthma. J Allergy Clin Immunol Pract. 2017; 5: 1572–1581.e3. 29122156 10.1016/j.jaip.2017.08.024

[149] US Food and Drug Administration. FDA labels for reslizumab (CINQAI^®^). 2019. (Accessed June 10, 2020). https://www.accessdata.fda.gov/drugsatfda_docs/label/2019/0761033s010lbl.pdf.

[150] European Medicines Agency. Assessment Report for reslizumab (CINQAERO^®^). 2019. (Accessed June 10, 2020). https://www.ema.europa.eu/en/documents/product-information/cinqaero-epar-product-information_en.pdf.

[151] Bernstein JA Virchow JC Murphy K Maspero JF Jacobs J Adir Y Humbert M Castro M Marsteller DA McElhattan J Hickey L Garin M Vanlandingham R Brusselle G Effect of fixed-dose subcutaneous reslizumab on asthma exacerbations in patients with severe uncontrolled asthma and corticosteroid sparing in patients with oral corticosteroid-dependent asthma: results from two phase 3, randomised, double-blind, placebo-controlled trials. Lancet Respir Med. 2020; 8: 461–474. 32066536 10.1016/S2213-2600(19)30372-8

[152] Castro M Wenzel SE Bleecker ER Pizzichini E Kuna P Busse WW Gossage DL Ward CK Wu Y Wang B Khatry DB van der Merwe R Kolbeck R Molfino NA Raible DG Benralizumab, an anti-interleukin 5 receptor α monoclonal antibody, versus placebo for uncontrolled eosinophilic asthma: a phase 2b randomised dose-ranging study. Lancet Respir Med. 2014; 2: 879–890. 25306557 10.1016/S2213-2600(14)70201-2

[153] Park HS Lee SH Lee SY Kim MK Lee BJ Werkström V Barker P Zangrilli JG Efficacy and safety of benralizumab for Korean patients with severe, uncontrolled eosinophilic asthma. Allergy Asthma Immunol Res. 2019; 11: 508–518. 31172719 10.4168/aair.2019.11.4.508 PMC6557768

[154] Liu W Ma X Zhou W Adverse events of benralizumab in moderate to severe eosinophilic asthma: A meta-analysis. Medicine (Baltimore). 2019; 98: e15868. 31145343 10.1097/MD.0000000000015868 PMC6709166

[155] US Food and Drug Administration. FDA labels for benralizumab (FASENRA). 2019. https://www.accessdata.fda.gov/drugsatfda_docs/label/2019/761070s005lbl.pdf (Accessed June 10, 2020).

[156] US Food and Drug Administration. FDA labels for benralizumab (FASENRA). 2019. https://www.accessdata.fda.gov/drugsatfda_docs/label/2019/761070s005lbl.pdf (Accessed June 10, 2020).

[157] Bourdin A Shaw D Menzies-Gow A FitzGerald JM Bleecker ER Busse WW Ferguson GT Brooks L Barker P Gil EG Martin UJ Two-year integrated steroid-sparing analysis and safety of benralizumab for severe asthma. J Asthma. 2019; 1–9. 10.1080/02770903.2019.1705333 31859541

[158] Ou Z Chen C Chen A Yang Y Zhou W Adverse events of Dupilumab in adults with moderate-to-severe atopic dermatitis: A meta-analysis. Int Immunopharmacol. 2018; 54: 303–310. 29182975 10.1016/j.intimp.2017.11.031

[159] European Medicines Agency. Assessment report of dupilumab (DUPIXENT^®^). 2019. https://www.ema.europa.eu/en/documents/variation-report/dupixent-hc-4390-x-0004-g-epar-assessment-report-extension_en.pdf.

[160] US Food and Drug Administration. FDA labels for dupilumab (DUPIXENT^®^). 2020. https://www.accessdata.fda.gov/drugsatfda_docs/label/2020/761055s020lbl.pdf.

[161] US Food and Drug Administration. FDA labels for lanadelumab (TAKHZYRO^®^). 2018. https://www.accessdata.fda.gov/drugsatfda_docs/label/2018/761090s001lbl.pdf. (Accessed June 9, 2020).

[162] European Medicines Agency. Assessment Report for lanadelumab (TAKHZYRO^®^). 2020. https://www.ema.europa.eu/en/documents/product-information/takhzyro-epar-product-information_en.pdf (Accessed June 9, 2020).

[163] Hanania NA Noonan M Corren J Korenblat P Zheng Y Fischer SK Cheu M Putnam WS Murray E Scheerens H Holweg CT Maciuca R Gray S Doyle R McClintock D Olsson J Matthews JG Yen K Lebrikizumab in moderate-to-severe asthma: pooled data from two randomised placebo-controlled studies. Thorax. 2015; 70: 748–756. 26001563 10.1136/thoraxjnl-2014-206719 PMC4515999

[164] Hanania NA Korenblat P Chapman KR Bateman ED Kopecky P Paggiaro P Yokoyama A Olsson J Gray S Holweg CT Eisner M Asare C Fischer SK Peng K Putnam WS Matthews JG Efficacy and safety of lebrikizumab in patients with uncontrolled asthma (LAVOLTA I and LAVOLTA II): replicate, phase 3, randomised, double-blind, placebo-controlled trials. Lancet Respir Med. 2016; 4: 781–796. 27616196 10.1016/S2213-2600(16)30265-X

[165] Korenblat P Kerwin E Leshchenko I Yen K Holweg CTJ Anzures-Cabrera J Martin C Putnam WS Governale L Olsson J Matthews JG Efficacy and safety of lebrikizumab in adult patients with mild-to-moderate asthma not receiving inhaled corticosteroids. Respir Med. 2018; 134: 143–149. 29413502 10.1016/j.rmed.2017.12.006

[166] Wollenberg A Howell MD Guttman-Yassky E Silverberg JI Kell C Ranade K Moate R van der Merwe R Treatment of atopic dermatitis with tralokinumab, an anti-IL-13 mAb. J Allergy Clin Immunol. 2019; 143: 135–141. 29906525 10.1016/j.jaci.2018.05.029

[167] Panettieri RA Sjöbring U Péterffy A Wessman P Bowen K Piper E Colice G Brightling CE Tralokinumab for severe, uncontrolled asthma (STRATOS 1 and STRATOS 2): two randomised, double-blind, placebo-controlled, phase 3 clinical trials. Lancet Respir Med. 2018; 6: 511–525. 29792288 10.1016/S2213-2600(18)30184-X

[168] Busse WW Brusselle GG Korn S Kuna P Magnan A Cohen D Bowen K Piechowiak T Wang MM Colice G Tralokinumab did not demonstrate oral corticosteroid-sparing effects in severe asthma. Eur Respir J. 2019; 53: 1800948. 30442714 10.1183/13993003.00948-2018

[169] Carlsson M Braddock M Li Y Wang J Xu W White N Megally A Hunter G Colice G Evaluation of antibody properties and clinically relevant immunogenicity, anaphylaxis, and hypersensitivity reactions in two phase III trials of tralokinumab in severe, uncontrolled asthma. Drug Saf. 2019; 42: 769–784. 30649752 10.1007/s40264-018-00788-w PMC6520328

[170] European Medicines Agency. Assessment report of secukinumab (COSENTYX^®^). 2015. http://www.ema.europa.eu/docs/en_GB/document_library/EPAR_-_Assessment_Report_-_Variation/human/003729/WC500199574.pdf (Accessed June 9, 2020).

[171] Blauvelt A Safety of secukinumab in the treatment of psoriasis. Expert Opin Drug Saf. 2016; 15: 1413–1420. 27545070 10.1080/14740338.2016.1221923

[172] Deodhar A Mease PJ McInnes IB Baraliakos X Reich K Blauvelt A Leonardi C Porter B Das Gupta A Widmer A Pricop L Fox T Long-term safety of secukinumab in patients with moderate-to-severe plaque psoriasis, psoriatic arthritis, and ankylosing spondylitis: integrated pooled clinical trial and post-marketing surveillance data. Arthritis Res Ther. 2019; 21: 111 31046809 10.1186/s13075-019-1882-2 PMC6498580

[173] US Food and Drug Administration. FDA labels for secukinumab (COSENTYX^®^). 2020. https://www.accessdata.fda.gov/drugsatfda_docs/label/2020/125504s031lbl.pdf (Accessed June 9, 2020).

[174] European Medicines Agency. Assessment Report secukinumab (COSENTYX^®^). 2020. https://www.ema.europa.eu/en/documents/product-information/cosentyx-epar-product-information_en.pdf (Accessed June 9, 2020).

[175] Grace E Goldblum O Renda L Agada N See K Leonardi C Menter A Injection site reactions in the federal adverse event reporting system (FAERS) post-marketing database vary among biologics approved to treat moderate-to-severe psoriasis. Dermatol Ther (Heidelb). 2020; 10: 99–106. 31734937 10.1007/s13555-019-00341-2 PMC6994575

[176] Nemoto O Furue M Nakagawa H Shiramoto M Hanada R Matsuki S Imayama S Kato M Hasebe I Taira K Yamamoto M Mihara R Kabashima K Ruzicka T Hanifin J Kumagai Y The first trial of CIM331, a humanized antihuman interleukin-31 receptor A antibody, in healthy volunteers and patients with atopic dermatitis to evaluate safety, tolerability and pharmacokinetics of a single dose in a randomized, double-blind, placebo-controlled study. Br J Dermatol. 2016; 174: 296–304. 26409172 10.1111/bjd.14207

[177] Silverberg JI Pinter A Pulka G Poulin Y Bouaziz JD Wollenberg A Murrell DF Alexis A Lindsey L Ahmad F Piketty C Clucas A Phase 2B randomized study of nemolizumab in adults with moderate-to-severe atopic dermatitis and severe pruritus. J Allergy Clin Immunol. 2020; 145: 173–182. 31449914 10.1016/j.jaci.2019.08.013

[178] Ständer S Yosipovitch G Legat FJ Lacour JP Paul C Narbutt J Bieber T Misery L Wollenberg A Reich A Ahmad F Piketty C Trial of nemolizumab in moderate-to-severe prurigo nodularis. N Engl J Med. 2020; 382: 706–716. 32074418 10.1056/NEJMoa1908316

[179] Chinthrajah S Cao S Liu C Lyu SC Sindher SB Long A Sampath V Petroni D Londei M Nadeau KC Phase 2a randomized, placebo-controlled study of anti-IL-33 in peanut allergy. JCI Insight. 2019; 4: e131347 10.1172/jci.insight.131347 PMC6948865 31723064

[180] Ghosh S Gensler LS Yang Z Gasink C Chakravarty SD Farahi K Ramachandran P Ott E Strober BE Correction to: Ustekinumab safety in psoriasis, psoriatic arthritis, and Crohn’s disease: an integrated analysis of phase II/III clinical development programs. Drug Saf. 2019; 42: 809 31012051 10.1007/s40264-019-00816-3 PMC6520476

[181] US Food and Drug Administration. FDA labels for ustekinumab (STELARA^®^). 2020. https://www.accessdata.fda.gov/drugsatfda_docs/label/2020/125261Orig1s153,761044s005lbl.pdf (Accessed June 9, 2020).

[182] European Medicines Agency. Assessment report of ustekinumab (STELARA^®^). 2020. https://www.ema.europa.eu/en/documents/product-information/stelara-epar-product-information_en.pdf (Accessed June 9, 2020).

